# The Effect of Guided Web-Based Cognitive Behavioral Therapy on Patients With Depressive Symptoms and Heart Failure: A Pilot Randomized Controlled Trial

**DOI:** 10.2196/jmir.5556

**Published:** 2016-08-03

**Authors:** Johan Gustav Lundgren, Örjan Dahlström, Gerhard Andersson, Tiny Jaarsma, Anita Kärner Köhler, Peter Johansson

**Affiliations:** ^1^ Division of Nursing Science Department of Social and Welfare Studies Linköping University Norrköping Sweden; ^2^ Disability Research Division Department of Behavioural Sciences and Learning Linköping University Linköping Sweden; ^3^ Psychology Department of Behavioural Sciences and Learning Linköping University Linköping Sweden; ^4^ Cardiovascular medicine, cardiology Department of Medical and Health Sciences. Department of Social and Welfare Studies Linköping University Linköping Sweden

**Keywords:** heart failure, depression, Internet-based cognitive behavioral therapy, cognitive behavioral therapy, Internet, eHealth

## Abstract

**Background:**

Depressive symptoms, and the associated coexistence of symptoms of anxiety and decreased quality of life (QoL), are common in patients with heart failure (HF). However, treatment strategies for depressive symptoms in patients with HF still remain to be established. Internet-based cognitive behavioral therapy (ICBT), as guided self-help CBT programs, has shown good effects in the treatment of depression. Until now, ICBT has not been evaluated in patients with HF with depressive symptoms.

**Objective:**

The aims of this study were to (1) evaluate the effect of a 9-week guided ICBT program on depressive symptoms in patients with HF; (2) investigate the effect of the ICBT program on cardiac anxiety and QoL; and (3) assess factors associated with the change in depressive symptoms.

**Methods:**

Fifty participants were randomized into 2 treatment arms: ICBT or a Web-based moderated discussion forum (DF). The Patient Health Questionnaire-9 was used to measure depressive symptoms, the Cardiac Anxiety Questionnaire (CAQ) was used to measure cardiac-related anxiety, and the Minnesota Living with Heart Failure questionnaire was used to measure QoL. Data were collected at baseline and at follow-up at the end of the 9-week intervention. Intention-to-treat analysis was used, and missing data were imputed by the Expectation-Maximization method. Between-group differences were determined by analysis of covariance with control for baseline score and regression to the mean.

**Results:**

No significant difference in depressive symptoms between the ICBT and the DF group at the follow-up was found, [F(1,47)=1.63, *P*=.21] and Cohen´s d=0.26. Secondary within-group analysis of depressive symptoms showed that such symptoms decreased significantly in the ICBT group from baseline to the follow-up (baseline M=10.8, standard deviation [SD]=5.7 vs follow-up M=8.6, SD=4.6, t(24)=2.6, *P*=.02, Cohen´s d=0.43), whereas in the DF group, there was no significant change (baseline M=10.6, SD=5.0, vs follow-up M=9.8, SD=4.3, t(24)=0.93, *P*=.36. Cohen´s d=0.18). With regard to CAQ and QoL no significant differences were found between the groups (CAQ [d(1,47)=0.5, *P*=.48] and QoL [F(1,47)=2.87, *P*=.09]). In the ICBT group in the CAQ subscale of fear, a significant within-group decrease was shown (baseline M=1.55 vs follow-up M=1.35, *P*=.04). In the ICBT group, the number of logins to the Web portal correlated significantly with improvement in depressive symptoms (*P*=.02), whereas higher age (*P*=.01) and male sex (*P*=.048) were associated with less change in depressive symptoms. This study is underpowered because of difficulties in the recruitment of patients.

**Conclusions:**

Guided ICBT adapted for persons with HF and depressive symptoms was not statistically superior to participation in a Web-based DF. However, within the ICBT group, a statically significant improvement of depressive symptoms was detected.

**ClinicalTrial:**

Clinicaltrials.gov NCT01681771; https://clinicaltrials.gov/ct2/show/NCT01681771 (Archived by WebCite at http://www.webcitation.org/6ikzbcuLN)

## Introduction

Depressive symptoms are common in patients with heart failure (HF), affecting about 20%-40% of the HF population [1-3]. They lead to higher morbidity and mortality and diminish self-care and health-related quality of life (QoL) [3]. However, treatment strategies for depressive symptoms in patients with HF still remain to be established [3,4].

HF has an unpredictable trajectory with disturbing and limiting symptoms that frequently change, leading to a shift between good and bad days [5] and with a constant risk of hospitalization or death [6,7]. Patients with HF may therefore be prone to developing negative thoughts, rumination, and feelings of hopelessness about loss of health and independence and an uncertain future [8], and this can lead to the development of depression.[9,10]. A vast majority of patients with HF and depressive symptoms also have symptoms of anxiety [11]. Depressive symptoms have a strong negative impact on QoL in patients with HF [3,4]. Because anxiety and depression are closely related, an intervention focusing on decreasing depression may also improve symptoms of anxiety and increase QoL. In general, depressive symptoms can be treated, either by psychotherapy or by pharmacology [12]. However, the impact of pharmacological treatment of depression in HF is not clear [4] and may be complicated due to an already complex pharmacological treatment regime [2]. Furthermore, patients with heart disease seem to prefer talking therapies such as cognitive behavioral therapy (CBT) over pharmacological treatment [8].

In CBT, patients become active participants in their treatment and perform tasks to become aware of and to modify negative thoughts and unhelpful behaviors. By developing skills to cope with these negative thoughts and behaviors, CBT also contributes to a decrease of negative emotions [13]. In HF patients, Freedland et al [14] demonstrated that undergoing CBT for 6 months decreased depression and Gary et al [15] found CBT to be beneficial, especially when combined with physical exercise. In these studies, CBT was provided face-to-face. Due to the lack of CBT therapists to deliver such face-to-face CBT, combined with the large number of HF patients with depression, most HF patients with depressive symptoms might not get access to CBT. Internet-based cognitive behavioral therapy (ICBT) may be an alternative to face-to-face CBT. ICBT has been shown to be a good and time-efficient method for the treatment of depressive symptoms and also effective when delivered by professionals other than psychotherapists. ICBT might, therefore, be considered as an attractive treatment strategy for depression in HF [16], but this is an area still waiting to be explored. Furthermore, since the frequency of participation in CBT treatment [17], level of depressive symptoms pre-intervention [18], age [19], sex, and New York Heart Association Class (NYHA class) [2] may impact changes in depressive symptoms, it is important to investigate these factors in intervention programs.

Recently our group showed that an ICBT program designed for HF patients was feasible [20], but the effect of ICBT on depressive symptoms in patients with HF has, to our knowledge, not been tested in a randomized controlled trial. The primary aim of this study was therefore to evaluate the short-term effect of ICBT on depressive symptoms in patients with HF. A second aim was to investigate the effect of the ICBT program on cardiac anxiety and QoL (secondary outcomes). A third aim was to assess these factors’ associations with the change in depressive symptoms.

## Methods

### Design

An open label, randomized control design was used.

### Recruitment Procedure and Inclusion

**Figure 1:**
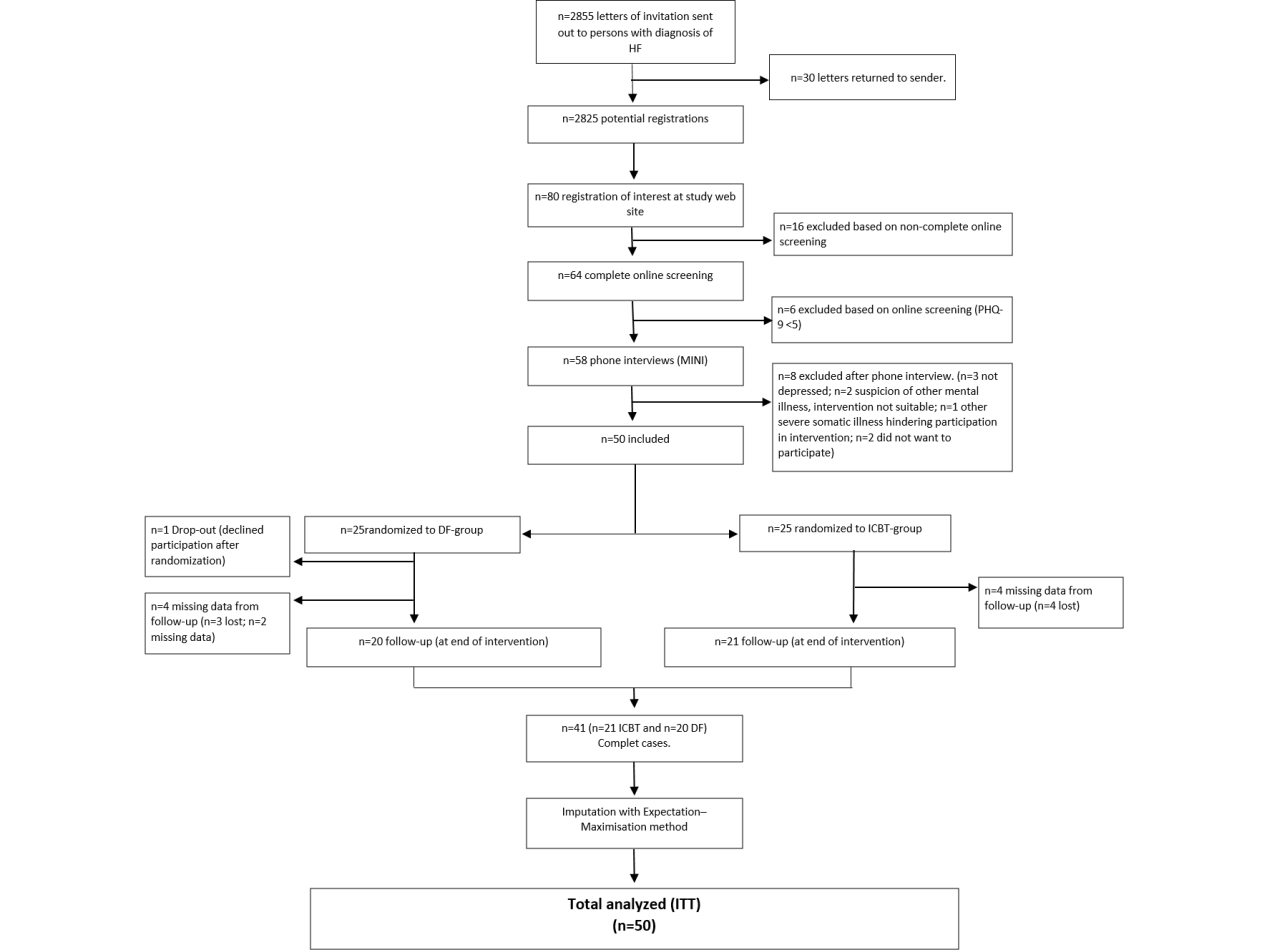
Flow diagram of participants. DF, discussion forum; HF, heart failure; ICBT, Internet-based cognitive behavioral therapy; PHQ-9, Patient Health Questionnaire-9.

To recruit participants, an information letter was sent to all patients who had an outpatient appointment or who had been admitted to hospital with the main diagnosis of HF during 2013 and 2014 in 4 hospitals in the southeast of Sweden (Figure 1). Inclusion criteria were at least mild depressive symptoms (the Patient Health Questionnaire-9 (PHQ-9)≥5), regular access to a computer with an Internet connection, access to a cellphone, being motivated to participate in treatment of depressive symptoms, and being aged older than 18 years. Exclusion criteria were suffering from other severe disease or illness that hindered participation in the study, admission to hospital during the last month due to HF, other treatment planned during the intervention that had been assessed as likely to hinder participation (such as surgery or planned inpatient treatment), severe level of depressive symptoms assessed as needing inpatient treatment, and high level of suicide risk or other psychiatric disorder assessed as making the intervention unsuitable. Patients who had perceived themselves as depressed or feeling down during (at least) the last 2 weeks and felt motivated and ready to participate in the study were invited to register their interest and perform a Web-based screening on the study website. Computer/Internet literacy was not a criterion for inclusion or exclusion. However, recipients interested in participation had to register on the study website and complete a Web-based screening form including an assessment of depressive symptoms by means of the PHQ-9, self-reported use of medication, comorbidities, NYHA class, and demographics.

A total of 64 patients completed the Web-based screening form and 58 were found to be possible candidates for inclusion. Candidates were contacted by telephone to check any uncertainties in the screening forms and to prevent multiple registrations. A structured phone assessment using the Mini International Neuropsychiatric Interview Swedish revised version 5.0.0 [21] was conducted to detect symptoms of other psychiatric health problems or suicidality that could hinder participation in the intervention, as shown in Figure 1. Two candidates were excluded because of suspicion of other mental illness and 3 candidates were excluded because they reported no depressive symptoms during the phone interview despite a screening PHQ-9 >4. During the phone call, the participant received detailed information about the study procedures. The 50 patients remaining after the telephone interview underwent baseline assessment and were randomized to either the ICBT group or the discussion forum group (DF group). Randomization was performed by a person blinded to screening and baseline data using Stata v.13 proc Ralloc with a block size of two. All participants gave written informed consent. No financial compensation was given to the participants. The regional ethical review board in Linköping, Sweden approved the study (dnr 2011/166-31). The study is registered at clinicaltrials.gov (NCT01681771).

### Intervention Procedure

Each participant received a password and a user name. Login to the Web portal (to access the treatment program, feedback, secure email, and assessment forms) required a 2-factor authentication system (requiring both a user name and password login and a single use code sent to a preregistered mobile phone) to protect sensitive information. If technical problems occurred, both the therapist and the participants could get support from a computer technician.

The ICBT program has been described in detail elsewhere [20]. Compared with the ICBT program tested in the proof-of-concept study and based on the findings that many of the participants in proof-of-concept study reported that the intervention was quite demanding in some parts, a short nonmandatory relaxation exercise was added in module 3. No changes were made to the program during the trial. To summarize, the ICBT program consisted of 7 consecutive modules that were worked with over 9 weeks (Table 1). Each module contained texts to be read and assignments to be completed by the participants. Written feedback was provided on each assignment. Participants could ask questions about the feedback or the content of the module using the secure email system. A mental health specialist nurse with experience of HF care provided feedback and answers within 24 hours during weekdays. The mental health nurse was supervised by a clinical psychologist and could consult a cardiologist and nurses specializing in HF care if needed. Participants who did not complete modules were reminded by personalized manually written emails, a maximum of 3 reminders were sent during a consecutive period of 2 weeks. Screenshots from different parts of the treatment platform are available (Multimedia Appendix 1), and a more comprehensive tour of the platform is available (Multimedia Appendix 2). Patients who were randomized to the DF group participated in a moderated discussion Web-based forum where new discussion topics were presented each week over a 9-week period. The topic was introduced without any extended background in a presentation consisting of statements and questions (Table 1). The discussion was performed in writing. Participants made their posts in discussion threads for each topic. To minimize waiting time, the participants in the DF group were allocated to 2 groups (n=12 and n=13) based on the dates they were enrolled in the study. All participants in the DF group were offered ICBT after the completion of the study.

**Table 1:** Overview of the guided Internet-based cognitive behavioral therapy program and the discussion forum.

Module	ICBT^a^ (content and CBT^b^ component)	DF^c^ (topic/question for discussion)	Week
1	Introduction (values and goals)	HF^d^: what do you know about HF?	1
2	Living with heart failure (psychoeducation)	The effect of HF on everyday life: do you have any tips you would like to share about handling HF?	2
3	Depression/depressive symptoms and heart failure (psychoeducation) Nonmandatory relaxation exercise	Self-care: do you have any methods that make self-care easier that you can share with the others in the DF?	3
4	Behavior activation 1: to enable change	Physical activity: have you been recommended physical activity? What is good or bad about physical activity when suffering from HF?	4
		Health care contacts: do you prepare yourself before health care appointments? Do you have any tips you can share with the others?	5
5	Behavior activation 2: to implement change	Health literacy: if you do not get answers from the health care system, do you look for information in other ways? Do you have any tips on where one can find information about health and diseases such as HF and depression?	6
		The effect of HF and depression on significant others: do you think that your health affects your relationships with others? If so, in what ways?	7
6	Problem solving: a tool for dealing with problems	The effect of HF and depression on significant others: how do you handle situations where your health affects other? Do you have any good examples of how to handle this that you can share?	8
7	Consummation	Summarizing: are there questions/topics that have not been discussed that you would like to address? How did you perceive the DF?	9

^a^ICBT: Internet-based cognitive behavioral therapy.

^b^CBT:cognitive behavioral therapy.

^c^DF:discussion forum.

^d^HF:heart failure.

### Measurements

Self-assessed data were collected on the Web at baseline (before the start of the intervention) and after the end of week 9 in the intervention. The data collection system for the follow-up was accessible for the participants from the 63rd day after the start of the intervention and could be completed during a 3-week period. All data except activity in the program was self-reported. Participants who did not complete outcome measures were reminded to do so by email up to 3 times.

### Depressive Symptoms (Primary Outcome Measurement)

Depressive symptoms were measured with the self-administered PHQ-9 [22]. The PHQ-9 is a 9-item instrument for measurement of depressive symptoms during the previous 2 weeks. Each item is answered on a 4-grade scale where zero means that the item does not affect the person, and scores 1 to 3 indicate that the item affects the person for periods ranging from several days to almost every day. The answers are summed to a total sum score in the range 0-27, with higher numbers representing a higher level of depressive symptoms. Proposed cutoff values are 0-4 for no depressive symptom, 5-9 for mild depression, 10-14 for moderate depression, 15-19 for moderately severe depression, and 20-27 for severe depression [23]. PHQ-9 has been tested for reliability and validity in patients with HF [24]. The Web-based version of PHQ-9 has demonstrated good interformat reliability [25]. Cronbach’s alpha of the PHQ-9 in this study was .81 (baseline) and .82 (follow-up).

### Cardiac Anxiety (Secondary Outcome Measurement)

The Cardiac Anxiety Questionnaire (CAQ) [26] was used to measure cardiac-related anxiety. CAQ is an 18-item self-rating instrument. Item scores range from 0 (never) to 4 (always). The total sum and mean total (range from 0 to 4) can be calculated for CAQ. The CAQ consists of 3 subscales: fear, avoidance, and heart-focused attention. CAQ has demonstrated good psychometric properties [26]. Cronbach’s alpha of the CAQ in this study was total scale .87 (baseline) and .85 (follow-up); subscale of fear .83 and .80; subscale of avoidance .89 and .88; subscale of heart-focused attention .69 and .70.

### QoL (Secondary Outcome Measurement)

QoL was measured with the disease-specific instrument Minnesota Living with Heart Failure questionnaire (MLHF) [27]. MLHF is a 21-item self-rating instrument. Each item is scored on a 6-point Likert scale (no, 0 to very much, 5). The total score is in the range 0-105, and a lower score indicates better QoL. The MLHF can be divided into physical and emotional factors. The reliability of MLHF has been reported as good [28,29]. A change of 5 points has been suggested as clinically important [30], Cronbach’s alpha of the MLHF in this study was total score .93 (baseline) and .93 (follow-up); physical .91 and .90; emotional .93 and .92.

### Activity in the ICBT Program

Activity in the program was calculated by the number of modules that the participants worked with (ie, the module had been assigned to the participant and the participant had done some activity related to the module, eg, handed in an assignment or posted messages regarding the module to the feedback provider; ICBT group only) as well as the number of logins to the Web portal during the 9-week period (both groups). Data concerning activity was aggregated from the Web portals log.

### Statistical Methods and Power Analysis

Analysis of participants’ characteristics was performed with descriptive statistics (mean, standard deviation, percent, and frequencies). For continuous variables, assumptions of normality were checked and primary outcome measurements were found suitable for parametric analysis. Analysis of covariance (ANCOVA) adjusting for baseline scores and regression to the mean [31] was used for comparison between groups (ie, ICBT vs DF). Paired samples *t* tests were used for within-group comparisons. Effect size was calculated with Cohen´s *d*. A small effect is considered to be between 0.2 and .0.5, a medium effect is considered between 0.5 and 0.8, and a value above 0.8 is considered to be a large effect. Pearson`s *r* or Kendall´s tau-b were used to analyze associations with change in the level of depressive symptoms. A chi-square test was used for nominal data, and if the expected number of observations was less than 5, Fisher´s exact test was used. Subtracting the baseline sum from the follow-up sum gave a figure indicating the change in level of depressive symptoms. A negative value meant a decrease of depressive symptoms, whereas a positive value meant an increase of depressive symptoms at the follow-up compared with baseline. All analyses were performed according to the intention-to-treat principle, regardless of actual completion of the ICBT program or DF.

A total of 18% (n=9) of the participants had missing data at the follow-up measurement. Missing values analysis was performed and data missing completely at random was assumed because there were no significant differences between background variables for participants with complete data versus incomplete data, and Little´s test for missing completely at random was not significant (χ^2^(111, N=50)=82.07, *P*=.98). Missing values were imputed using the Expectation-Maximization (EM) method. Based on observed values, EM imputes missing values based on maximum likelihood estimates in an iterative process [32]. Subgroup analysis was performed on participants with complete data. Power analysis showed that a total of 104 participants were needed (effect size=0.5, alpha=.05 (*Z*=1.96), power 0.80 (*Z* −0.84). Statistical analysis was performed using IBM SPSS, version 23 and Microsoft Excel 2013. *P* value <.05 was considered as significant.

## Results

The characteristics of the participants are presented in (Table 2). Most (n=29, 59%) of the participants were men, and the mean age was 63 years (range 23-80). Participants in the DF group reported significantly more prescription of diuretics (χ^2^(1, N *=* 50)=4.67, *P*=.03) and sleep medications (χ^2^(1, N=50)=3.95, *P*=.047). Participants who did not complete the follow-up assessment (n=9) did not significantly differ at baseline in level of depression (PHQ-9 *t* (48)=1.89, *P*=.07), cardiac-related anxiety (CAQ *t* (48)=-0.55, *P*=.60), or QoL (MLHF *t* (48)=0.69, *P*=.50) from those who completed the assessment.

**Table 2:** Participants’ characteristics.

				Total (n=50)	ICBT group (n=25)	DF group (n=25)
Demographics						
			Age, M (SD)	62.9 (12.8)	63.6 (13.9)	62.3 (11.7)
			Men, n (%)	29 (59)	15 (60)	14 (58)
			Cohabitation^a^, n (%)	37 (76)	19 (76)	18 (75)
			Level of depression at screening PHQ-9, M (SD)	11.5 (4.8)	11.8 (4.4)	11.2 (5.2)
HF^b^symptoms and treatment						
			NYHA^c^ class, n (%)			
			I	11 (22)	8 (32)	3 (12)
			II	20 (40)	12 (48)	8 (32)
			III	18 (36)	5 (20)	13 (52)
			IV	1 (2)	0 (0)	1 (4)
			Dyspnea^d^, n (%)	48 (96)	24 (96)	23 (92)
			Fatigue^d^, n (%)	49 (98)	25 (100)	24 (96)
			Swollen legs or feet^d^, n (%)	23 (46)	14 (56)	12 (48)
			Time with HF>6 month/<6 month, n (%)	45/5 (88/10)	22/3 (88/12)	23/2 (92/8)
			Previously hospitalized due to HF, n (%)	36 (72)	17 (68)	19 (76)
			Beta blocker, n (%)	44 (88)	22 (88)	22 (88)
			ACE-I^e^/ARB^f^, n (%)	47 (94)	22 (88)	25 (100)
			Diuretics, n (%)	34 (68)	14 (56)	20 (80)^g^
Comorbidities, n (%)						
			Ischemic heart disease	18 (36)	8 (32)	10 (40)
			Hypertension	26 (52)	11 (44)	15 (60)
			Arrhythmia	26 (52)	14 (56)	12 (48)
			Diabetes	7 (14)	2 (8)	5 (20)
			Pulmonary disease	6 (12)	1 (4)	5 (20)
			Stroke or TIA	11 (22)	4 (16)	7 (28)
			Kidney disease	1 (2)	1 (4)	0 (0)
			Cancer	5 (10)	3 (12)	2 (8)
			Other psychiatric disorder^h^	2 (4)	2 (8)	0 (0)
Pharmacological antidepressive, anxiolytic, or sleep medication						
			Antidepressives	9 (18)	9 (12)	6 (24)
			Anxiolytics	2 (4)	1 (4)	1 (4)
			Sleep medication	14 (28)	4 (16)	10 (40)^g^

^a^Cohabitation includes participants that live with someone in a long-term relationship (including married). Not living with partner includes participants who were divorced, with partner deceased or living alone.

^b^HF, heart failure.

^c^NYHA, New York Heart Association.

^d^Symptoms reported to affect the participant very severely to little have been collapsed and reported as presence of symptoms.

^e^ACE-I, angiotensinogen-converting enzyme inhibitor.

^f^ARB, angiotensin receptor blocker.

^g^Significant difference between CBT and discussion groups (*P*<.05).

^h^Self-reported: anxiety disorder (n=1) and drug dependence (n=1).

### Primary Outcome: Level of Depressive Symptoms

**Figure 2:**
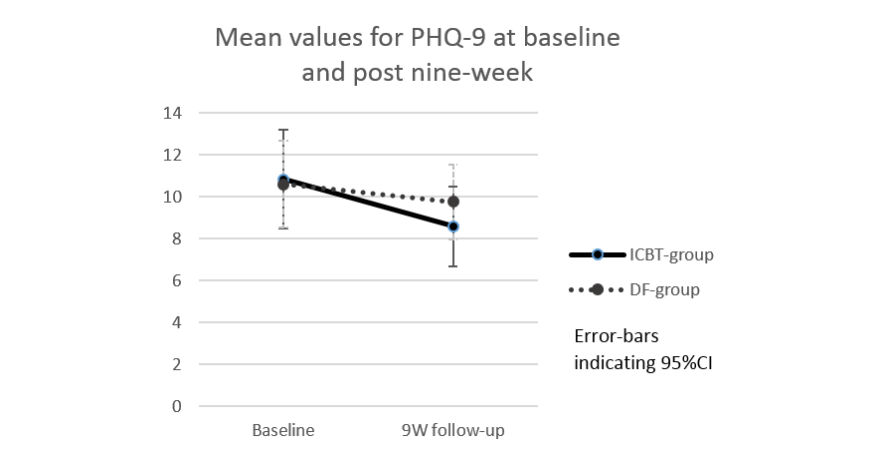
Mean values for PHQ-9 at baseline and follow-up in the 2 groups (n=25 ICBT and n=25 DF). DF, discussion forum; ICBT, Internet-based cognitive behavioral therapy; PHQ-9, Patient Health Questionnaire-9.

In the primary ANCOVA analysis, there was no significant difference in depressive symptoms between the ICBT and the DF group at the follow-up [*F* (1,47)=1.63, *P*=.21] Cohen´s *d=* 0.26. Secondary within-group analysis showed that depressive symptoms in patients in the ICBT group decreased significantly from baseline to the follow-up (Figure 2) (baseline M *=* 10.8, SD=5.7 vs follow-up M=8.6, SD=4.6, *t* (24)=2.6, *P*=.02. Cohen´s *d*=0.43). In the patients in the DF group, a small nonsignificant change in depressive symptoms was found (baseline M *=* 10.6, SD=5.0, vs follow-up M *=* 9.8, SD=4.3, *t* (24)=0.93, *P*=.36. Cohen´s *d=* 0.18).

### Secondary Outcomes: Cardiac-Related Anxiety and QoL

**Figure 3:**
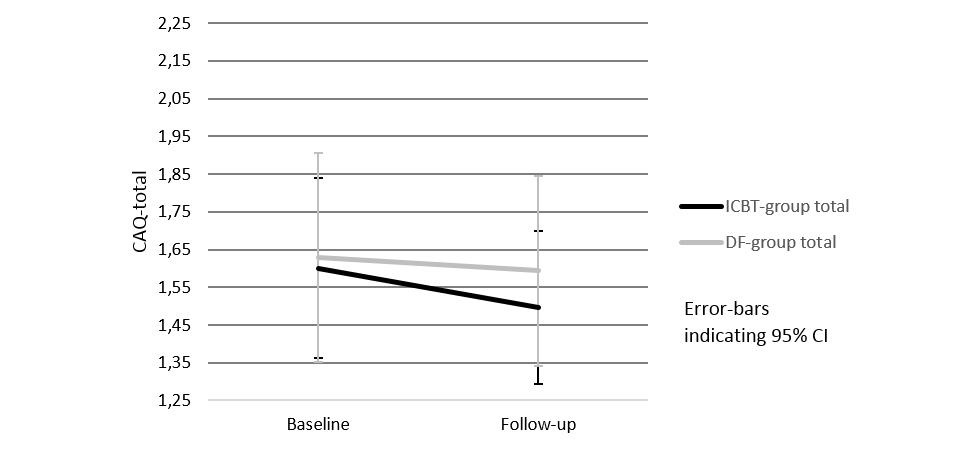
Change in cardiac anxiety—mean values for subscale of fear in the 2 groups (n=25 ICBT and n=25 DF). ANCOVA: [F(1,47)=1.57, *P*=.22], total scale range for CAQ (mean values): 0-4. ANCOVA, analysis of covariance; CAQ, Cardiac Anxiety Questionnaire; DF, discussion forum; ICBT, Internet-based cognitive behavioral therapy.

Between group comparison (ie, ANCOVA, ICBT vs DF) showed no statistically significant difference in the CAQ total score [F(1,47)=0.51, *P*=.48], Cohen´s *d=* 0.18, subscale of fear [*F* (1, 47)=1.57, *P*=.22], Cohen´s *d*=0.43, subscale of avoidance [F(1,47)=0.11, *P*=.74], Cohen´s *d=* 0.17, and subscale of heart-focused attention [F(1,47)=0.39, *P*=.54], Cohen´s *d=* 0.08. In the secondary within-group analysis, the ICBT group showed a decrease in the total CAQ score and in the subscale of fear. The decrease in the subscale of fear was statistically significant (baseline M=1.55, SD=0.73 vs follow-up M=1.35, SD=0.60, *t* (24)=2.18 *P*=.04. Cohen´s *d*=0.30), see Figure 3, but the decrease in the total score was not significant (baseline M=1.60, SD=0.58 vs follow-up M=1.49, SD=0.49, *t* (24)=1.25, *P*=.22. Cohen´s *d=* 0.31). In the subscales of avoidance and heart-focused attention, no significant changes were found (Multimedia Appendix 3). In the DF group, no significant changes in any of the CAQ scales were found (total *P*=.86, fear *P*=.92, avoidance *P*=.82, heart-focused attention *P*=.83).

Between-group analysis (ie, ANCOVA, ICBT vs DF) of MLHF revealed no significant differences for the total score [*F* (1,47)=2.87, *P*=.09], Cohen´s *d=* 0.51, the physical factor [*F* (1,47)=3.35, *P*=.07], Cohen´s *d=* 0.56, and the emotional factor [F(1,47)=0.20, *P*=.66], Cohen´s *d=* 0.37. The change in scores from baseline to the follow-up for the total score and the physical and emotional factors in the MLHF is shown in Multimedia Appendix 4. In the ICBT group, the mean total score decreased by 6.0 points, by 2.4 points in the physical factor and by 0.3 points in the emotional factor. None of the differences were statistically significant; total score baseline M=41.8, SD=20.5 vs follow-up M=35.8, SD=15.3, *t* (24)=1.79, *P*=.09, Cohen´s *d=* 0.33; the physical factor, baseline M=17.5, SD=8.7 vs follow-up M *=* 15.1, SD=7.5, *t* (24)=1.62, *P*=.12, Cohen´s *d=* 0.28; baseline M=10.8, SD *=* 7.2 vs follow-up M *=* 10.5, SD *=* 6.4, *t* (24)=0.31, *P*=.76, Cohen´s *d=* 0.05. In the DF group, the mean total score decreased by 1.9 points, by 0.2 points in the physical factor, and by 0.8 points in the emotional factor. None of the differences were significant; baseline M=47.1, SD=24.0 vs follow-up M=45.3 SD=21.3, *t* (24)=0.64, *P*=.53, Cohen´s *d=* 0.08; the physical factor baseline M=20.0, SD=10.6 vs follow-up M *=* 19.8, SD=8.9, *t* (24)=0.14, *P*=.89, Cohen´s *d=* 0.02; the emotional factor baseline M=13.7, SD *=* 6.3 vs follow-up M *=* 12.9, SD *=* 5.6, *t* (24)=0.91, *P*=.37, Cohen´s *d=* 0.14.

### Relationship Between Factors and Changes in Depressive Symptoms

The median number of modules performed in the ICBT group was 4. Six (24%) of the participants in the ICBT group had worked with all 7 modules and 15 (60%) had worked with at least 4 modules (ie, 57% of the program). There was no significant relationship between the number of modules completed and the change in depressive symptoms (τ_b_=.13, *P*=.46). In the ICBT group, the number of logins to the Web portal was significantly related to the change in depressive symptoms (ie, improvement in depressive symptoms) (*r*=−.50, *P*=.02) a similar relationship was found in the DF group although not as strong and not significant (*r*=−.32, *P*=.17). Age had a negative correlation with number of logins in the ICBT group (*r*=−.67, *P*<.001). In the DF group, this correlation was not so strong and not significant (*r=* −.24, *P*=.25).

The level of depressive symptoms at screening was not associated with the level of depressive symptoms at the follow-up. A separate analysis of participants with PHQ-9 ≥10 at screening (ICBT n=18, DF n=15) showed no significant difference between groups in the level of depressive symptoms at the follow-up [*F* (1,29)=1.30, *P*=.26] nor of participants with PHQ-9 ≤15 (ICBT n=19, DF n=19) [*F* (1,34)=0.82, *P*=.37]. Higher age correlated significantly with less change in depressive symptoms (*r*=.54, *P*=.01) in the ICBT group and women (n=8, complete cases) (M *=* −3.4, SD=4.6) had a significantly higher mean change in depressive symptoms compared with men (n=13, complete cases) (M=−0.08, SD=2.6) *t* (19)=2.12, *P*=.048. Cohen´s *d=* 0.89. The severity of HF as assessed by NYHA class was not associated with a change in depressive symptoms.

## Discussion

### Principal Findings

To our knowledge, this is the first study evaluating an ICBT program aimed at decreasing depressive symptoms in patients with HF. The recruitment of participants was more difficult than expected. Based on a prevalence of depressive symptoms among HF patients at approximately 20% [1], we expected about 571 of the 2852 contacted the patients have a significant level of depressive symptoms. However, only 80 patients registered as interested and 50 were found to be eligible for inclusion. Other studies of CBT in HF also appear to have difficulty in achieving sample sizes corresponding to power analysis [14,15,33]. Due to practical and economic reasons, we chose to end the study without achieving the targeted sample size. Other recruitment strategies may be more effective. However, there may also be a structural problem in reaching out to patients with HF and depressive symptoms. This is because depressive symptoms can reduce patients’ decision-making process due to ambivalence and impaired cognitive functioning [34]. Our primary analysis could not detect any significant difference in the reduction of depressive symptoms between the ICBT and DF groups; this may be explained by the slight reduction of depressive symptoms in the DF group. Dekker et al [35] reported similar results where depressive symptoms decreased in HF patients who received a brief CBT intervention or received standard care before discharge from hospital. The within-group analysis of depressive symptoms showed a significant decrease in the ICBT group but not in the DF group. These findings are in line with previous studies reporting that CBT for depression is significantly superior compared with no treatment, but only nominally superior compared with psychological placebo [33,36] such as the DF in our study. Studies specifically on HF patients with depressive symptoms also suggest that an active intervention, such as CBT or placebo with attention control such as DF can reduce depressive symptoms to a greater extent than standard care [14,15,37]. Designing a new ICBT program is a complex process [38]. Our program is to our knowledge one of the first ICBT programs for patients with HF and depressive symptoms. Furthermore, HF patients are often older compared with other patient groups treated with ICBT. Therefore, there is a need for more research to gain knowledge on how to design or redesign ICBT programs for the HF population. A possible future approach to achieve optimal results in treatment of depressive symptoms in HF could be a stepped care model [39] where patients could start with a type of DF or physical exercise, and if the depressive symptoms did not improve, ICBT could be added. However, such models have to be evaluated in further studies.

The secondary outcomes of CAQ and MLHF did not show any significant difference between the ICBT group and the DF group. However, in the ICBT group, a lower cardiac-related anxiety in the subscale of fear and an increased QoL was found in the within-group analysis. The increase in QoL of 6 points in the total MLHF score was not statistically significant; however, a change of 5 points in MLHF has been proposed as a measure of a clinically important change [30].

It is common for depressive symptoms in patients with HF to coexist with anxiety [11]. Anxiety CBT treatment can have a better effect on depressive symptoms than CBT for depression in patients with HF [40]. Thus, anxiety and worries may need special attention when designing or redesigning ICBT interventions for patients with HF and depressive symptoms.

We also found that the age and sex of the participant may need to be taken into account. Higher age and male sex correlated with less change in depressive symptoms in the ICBT group. Older people to some extent seem to benefit from CBT [20,41]; however, the evidence of benefit for them is greater in problem-solving therapy [42,43]. Our ICBT program relied to a large extent on behavioral activation and to a lesser extent on problem-solving therapy [20]. Most patients with HF are older and vulnerable, which raises an important question of whether problem-solving therapy should be used to a greater extent in future studies, as proposed by Alexopoulos et al [44]. Women showed more positive effects compared with men in the ICBT group. This is in line with the results of other ICBT studies [45,46]. However, sex as a predictor of outcome of CBT delivered by other modalities has shown inconsistent results; men have been reported to have a better response in telephone and face-to-face CBT [47]. More research is needed to determine whether CBT and ICBT should be adapted to the different sexes.

The cornerstone of CBT is to encourage participants and involve them in the treatment [48]. We found that activity in the program, as indicated by the number of logins, correlated significantly with a change in depressive symptoms. This suggests that helping participants in ICBT programs to be active is important for a positive outcome. In our program, participants who did not follow the pace of the program were reminded to do so by email. In contrast, others have used more intensive reminder techniques of both text messages and phone calls, and thereby achieved a higher adherence to ICBT treatment [49,50]. This indicates that more direct reminders can motivate less active participants. The number of modules performed did not correlate with level of depression at follow-up. The reasons for this have not been investigated in this study. However, during the study, we experienced that some patients early in the program reported that they felt better and therefore did not proceed with the next module. This may have affected the result negatively, thus one may speculate that patients might have improved even more if they had completed the program. Our study has shown that ICBT for HF patients with depressive symptoms is feasible. However, further research is needed to develop effective ICBT programs for depressive symptoms in HF patients. Furthermore, this study only evaluated the effect directly after the intervention; thus, the long-term effects of ICBT on depression in patients with HF need to be evaluated in future studies.

The generalizability of the results is limited for several reasons. One major limitation of our study is that it is underpowered. Post hoc power calculation for this study showed a power of 16% and a need for 462 patients to be included to achieve a statistically significant result as regards depressive symptoms. A reason for the need for such a large sample could be floor effects, because patients with mild depression were also included (PHQ-9≥5). A reason for including these patients is that even mild depression has a strong negative impact on QoL in HF patients [51]. The mean age in the study sample was lower compared with the HF population in the community (approximately 78 years) [52]; thus, it is unclear how the program works in older HF patients. Furthermore, the study could only include patients with access to a computer and the Internet, and therefore, the results cannot be generalized to patients without such access. On the other hand, in Sweden, 42% (n=355 793) of the population aged older than 75 years use computers and the Internet at home. This figure is expected to rise to approximately 80% in the coming 5-10 years [53]. There is a potential limitation in relying on self-reported data. However, all patients were identified by diagnostic codes for HF from electronic hospital records and were contacted by telephone to verify their reported medication and comorbidities. There were twice as many participants taking an antidepressant medication in the DF group compared with the ICBT group. The reason for this not being statistically significant is probably due to low power in the study. Subgroup analysis in this study has to be interpreted with caution due to the limited sample size. Missing data were imputed using the EM method. Although Littles´ test indicated that the missing data in our study appear to be missing at random, this can never be certain, and missing data and imputation can carry a risk of bias. Initially, all analysis was performed on both nonimputed data and imputed data, and we found no significant differences between the analysis with the exception of the ANCOVA on the physical factor of QoL, which presented a significant difference between the groups on nonimputed data but not on imputed data. There were more patients reporting use of antidepressive medication (nonsignificant) and sleep medication (significant) in the DF group. We do not think that this had any major impact on our result. For example, pharmacological antidepressive treatment in HF patients has shown a poor effect on depression [37]. The lack of power of this study may also have resulted in differences not being detected between participants completing the intervention and dropouts. Nevertheless, we think that our study is important since interventions for patients with HF and depressive symptoms are not widely studied. To our knowledge, our study is one of the first to investigate the effect of ICBT in HF and depressive symptoms. Development of new interventions is an iterative process [38] and although clear-cut results may be desirable, the novelty of the research area makes this unlikely. With our study’s limited sample size and its recruitment difficulties, the results may best serve as a foundation for further research rather than as clinical recommendations.

### Conclusion

Guided ICBT adapted for persons with HF and depressive symptoms was not statistically superior to participation in a Web-based DF. However, within the ICBT group, a statically significant improvement of depressive symptoms was detected.

## Appendix

Multimedia Appendix 1 Screenshots of the treatment platform.

Multimedia Appendix 2 Video of treatment platform.

Multimedia Appendix 3 Change in cardiac anxiety—mean values for total score and subscales of avoidance and heart-focused attention in the 2 groups (n=25 ICBT and n=25 DF).

Multimedia Appendix 4 Change in Minnesota Living with Heart Failure (MLHF)—total score and factors in the 2 groups (n=25 ICBT and n=25 DF).

Multimedia Appendix 5 CONSORT-EHEALTH Checklist V1.6.

## Abbreviations

CAQ: Cardiac Anxiety Questionnaire.
CBT: cognitive behavioral therapy.
DF: discussion forum.
EM: Expectation-Maximization.
HF: heart failure.
ICBT: Internet-based cognitive behavioral therapy.
MLHF: Minnesota Living with Heart Failure questionnaire.
PHQ-9: Patient Health Questionnaire-9.
QoL: quality of life.
