# Supplementary material for: The Effect of Guided Web-Based Cognitive Behavioral Therapy on Patients With Depressive Symptoms and Heart Failure: A Pilot Randomized Controlled Trial
Source: J Med Internet Res. 2016 Aug 3;18(8):e194. doi: 10.2196/jmir.5556 (PMC5070581; doi:10.2196/jmir.5556)
Supplement: Multimedia Appendix 1 [file jmir_v18i8e194_app1.pptx]

## Slide 1
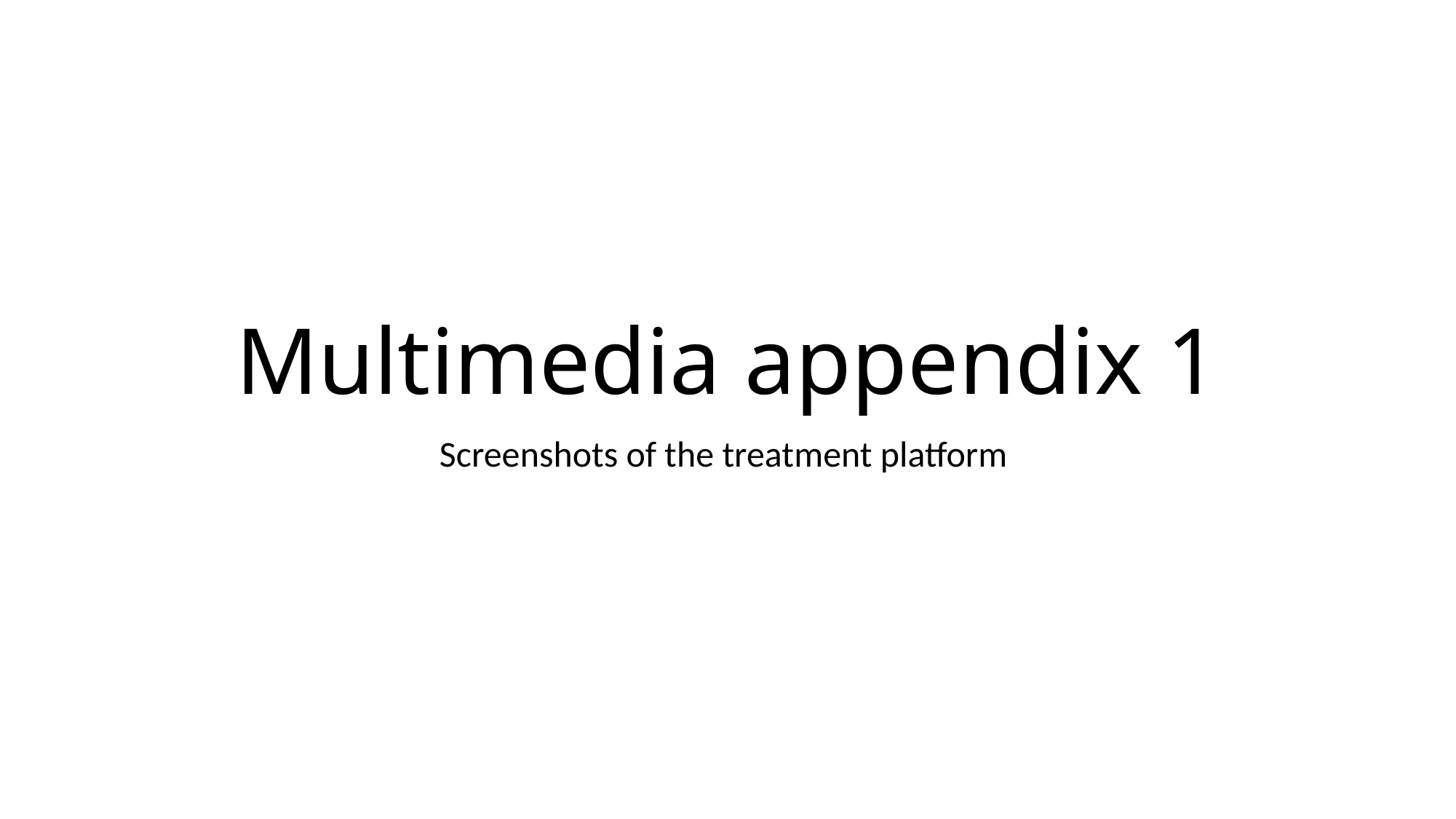

# Multimedia appendix 1
Screenshots of the treatment platform

## Slide 2
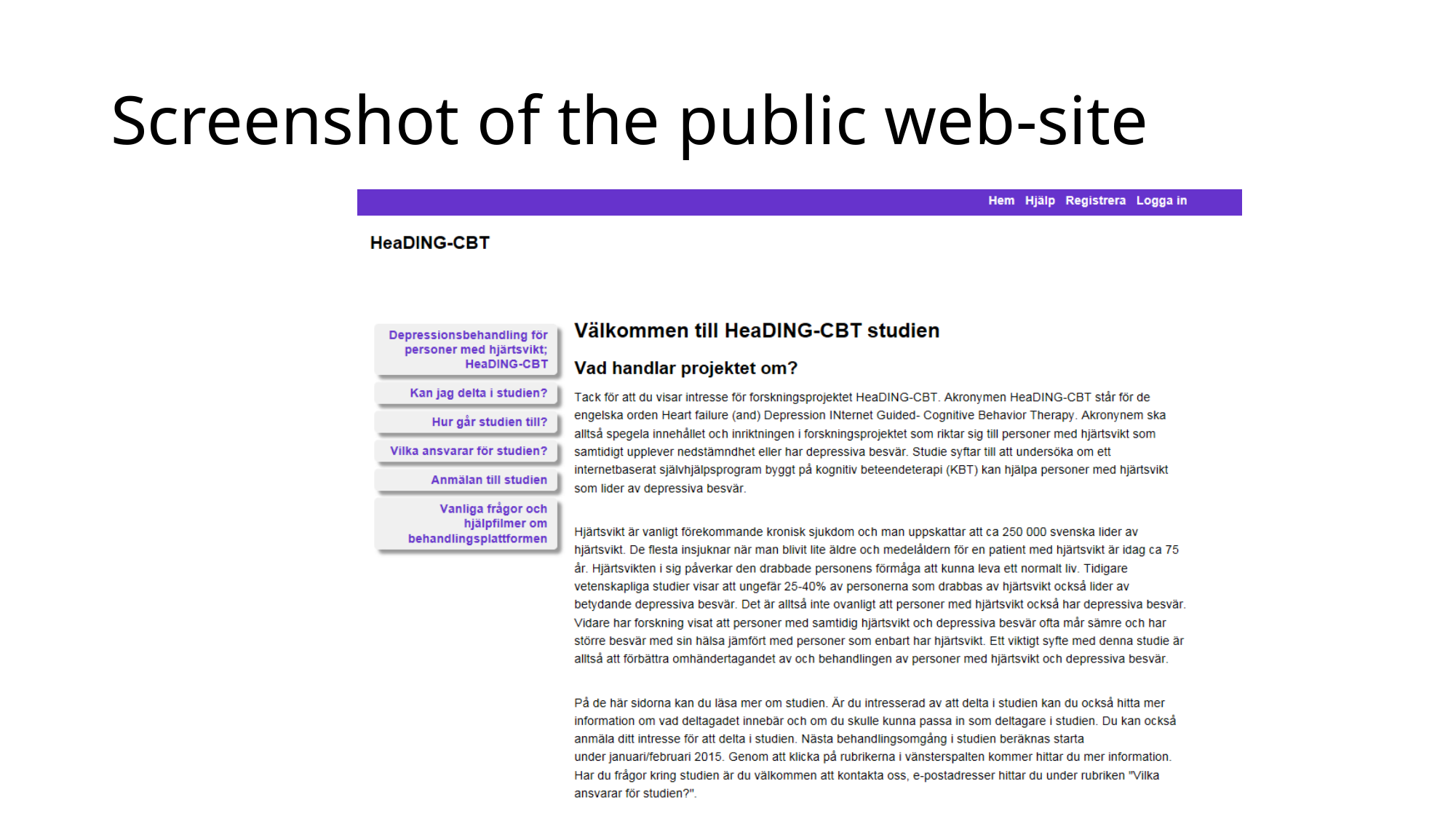

# Screenshot of the public web-site

## Slide 3
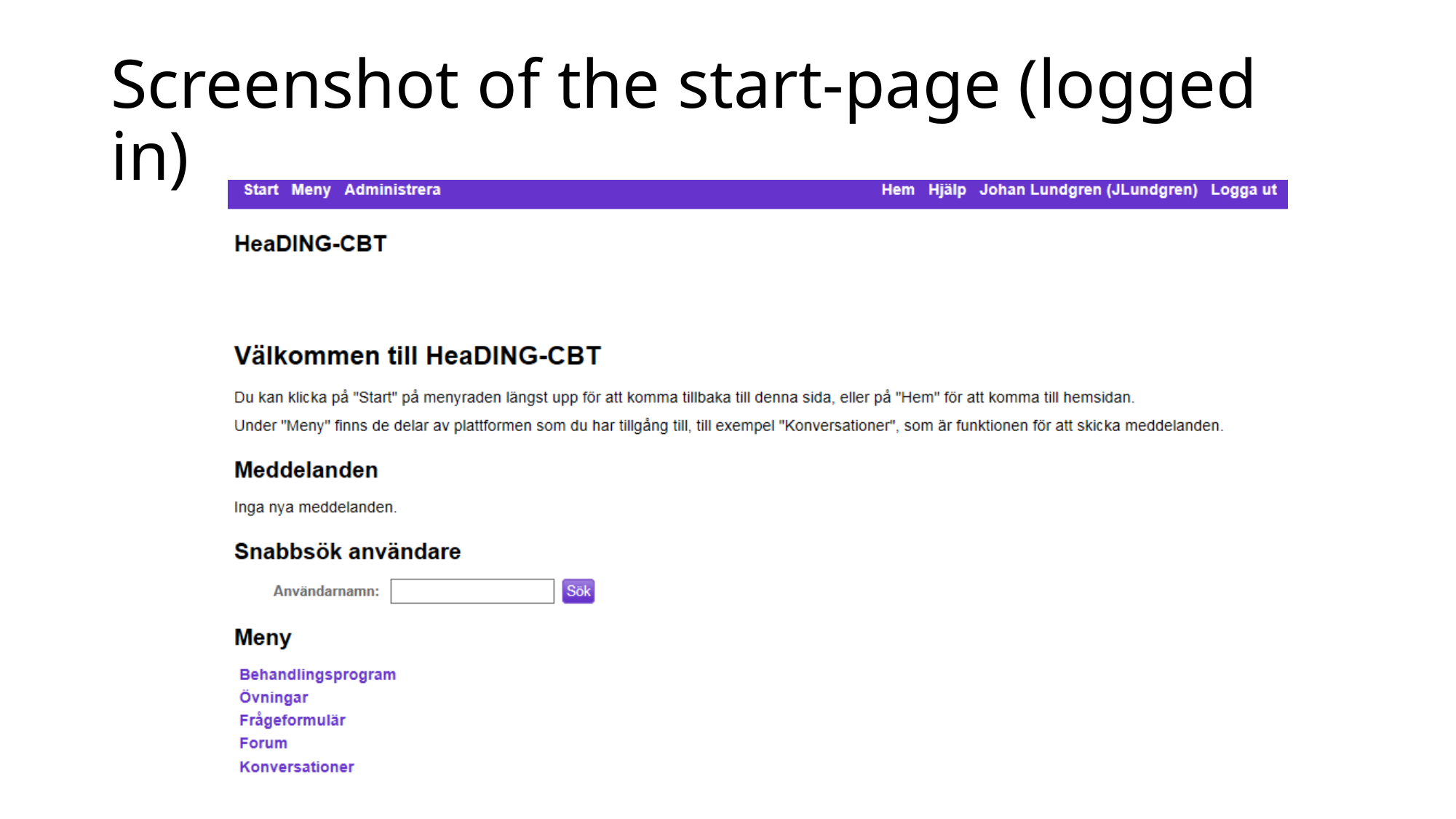

# Screenshot of the start-page (logged in)

## Slide 4
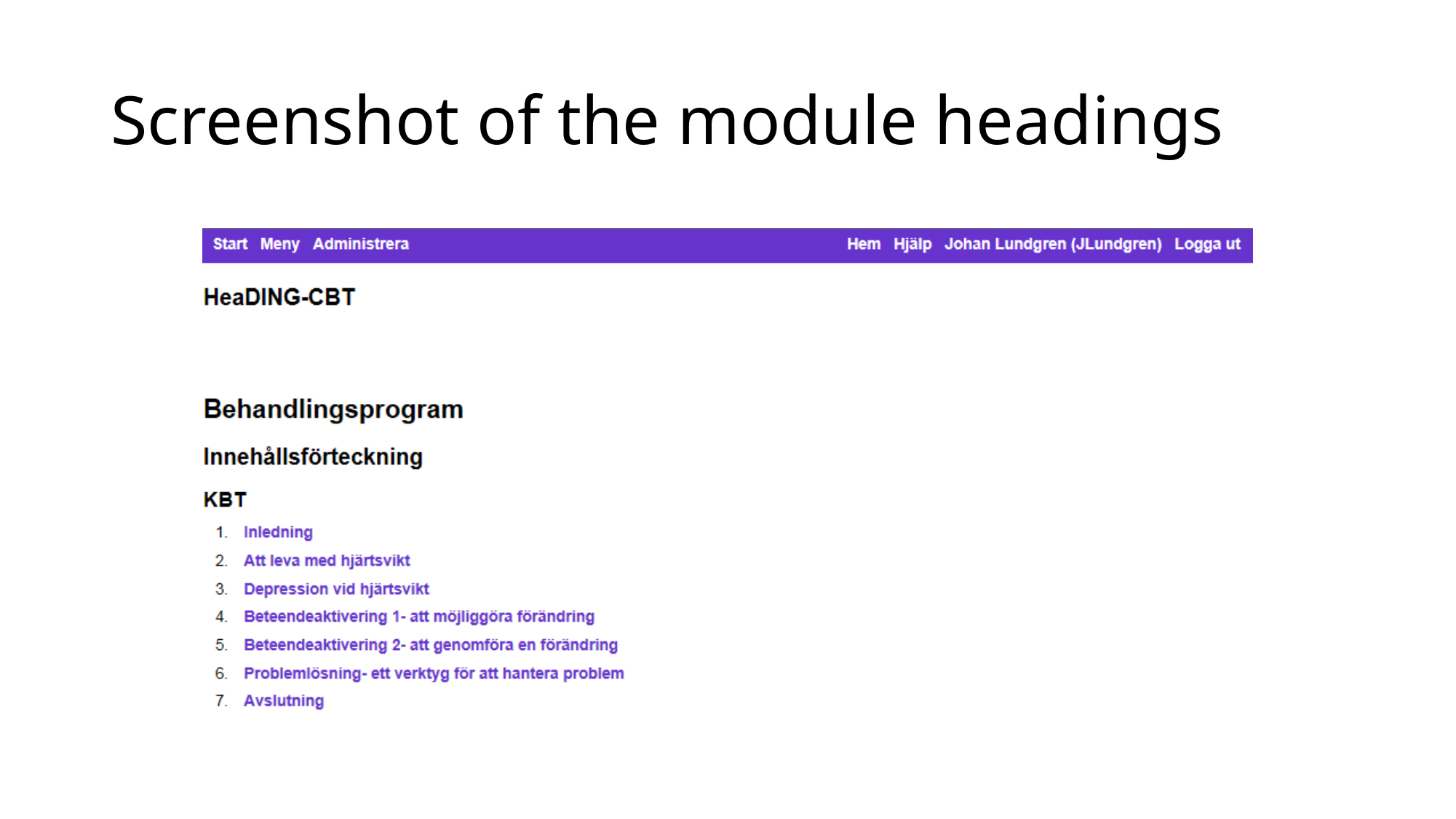

# Screenshot of the module headings

## Slide 5
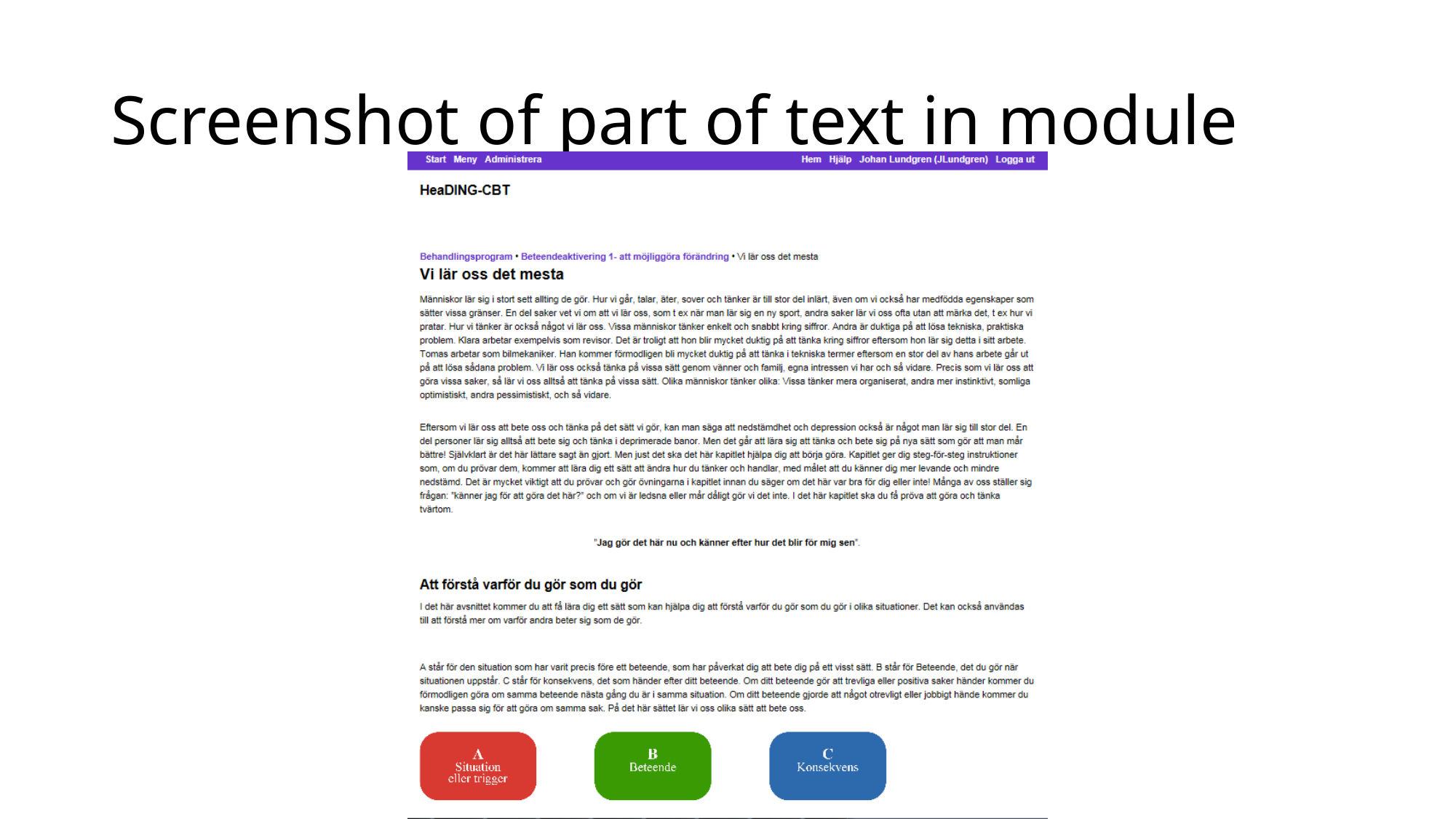

# Screenshot of part of text in module

## Slide 6
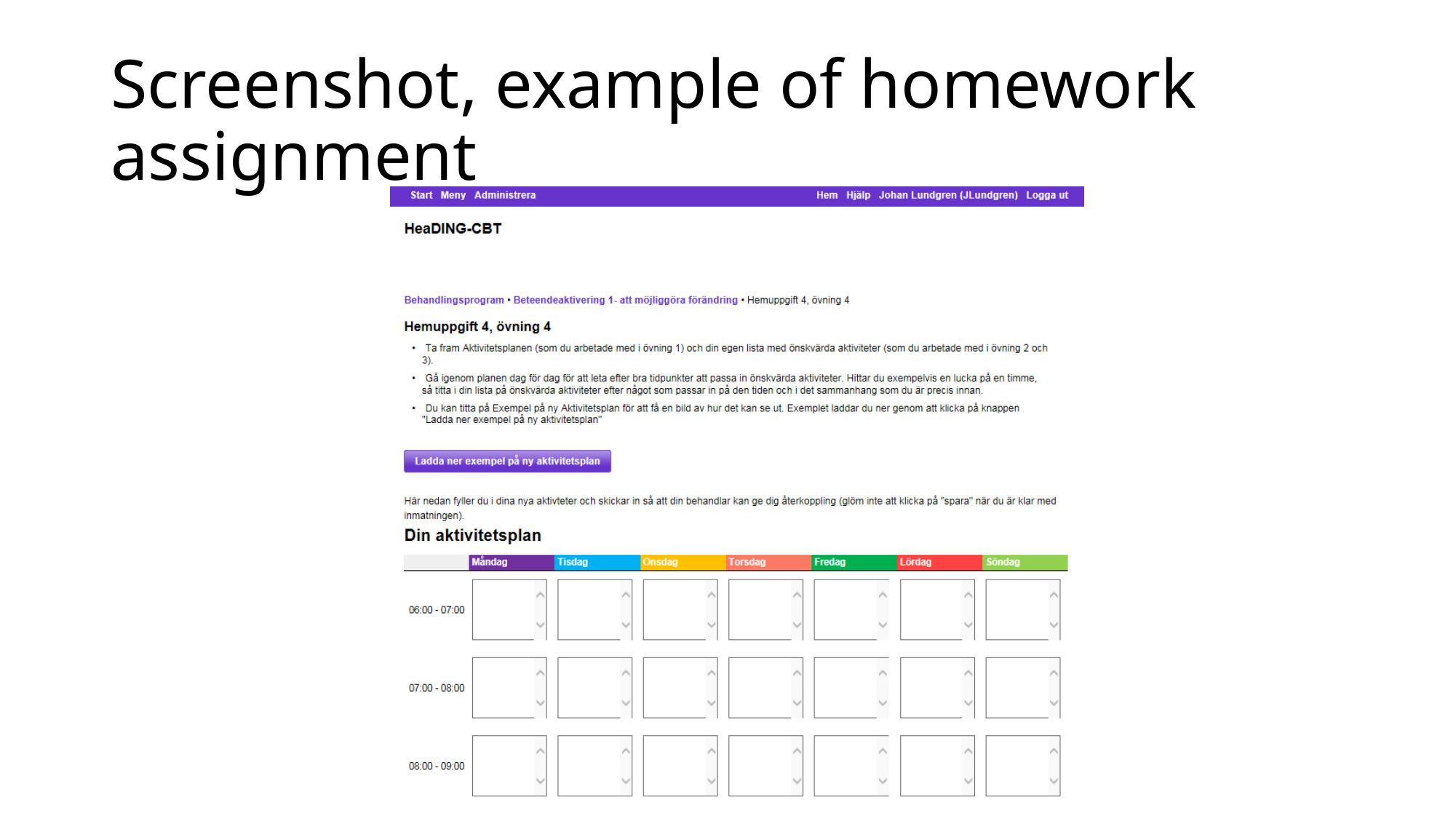

# Screenshot, example of homework assignment
