# Supplementary material for: The Effect of Guided Web-Based Cognitive Behavioral Therapy on Patients With Depressive Symptoms and Heart Failure: A Pilot Randomized Controlled Trial
Source: J Med Internet Res. 2016 Aug 3;18(8):e194. doi: 10.2196/jmir.5556 (PMC5070581; doi:10.2196/jmir.5556)
Supplement: Multimedia Appendix 2 [file jmir_v18i8e194_app2.pptx]

## Slide 1
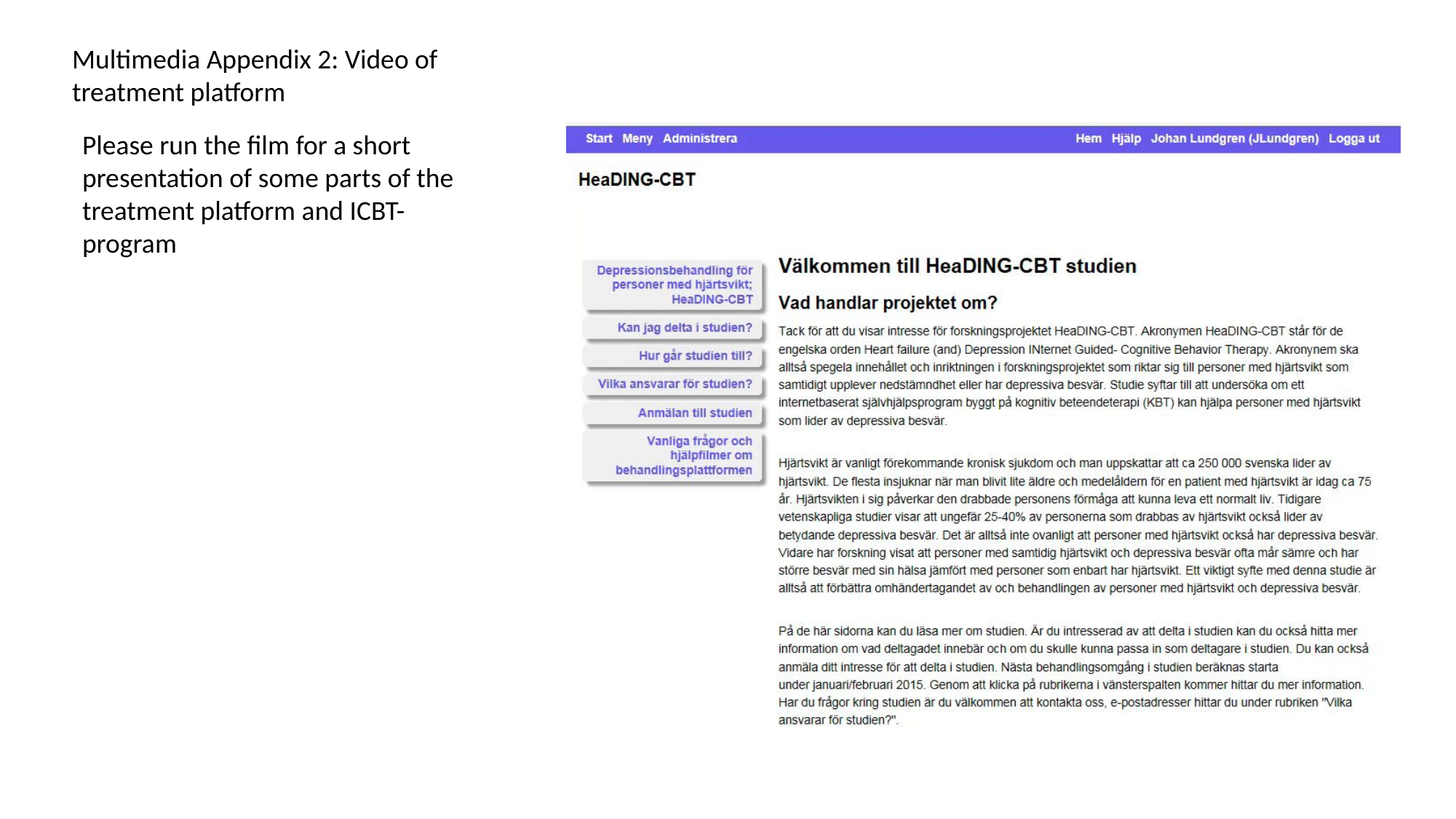

Multimedia Appendix 2: Video of treatment platform
Please run the film for a short presentation of some parts of the treatment platform and ICBT-program
