# Supplementary material for: The Effect of Guided Web-Based Cognitive Behavioral Therapy on Patients With Depressive Symptoms and Heart Failure: A Pilot Randomized Controlled Trial
Source: J Med Internet Res. 2016 Aug 3;18(8):e194. doi: 10.2196/jmir.5556 (PMC5070581; doi:10.2196/jmir.5556)
Supplement: Multimedia Appendix 3 [file jmir_v18i8e194_app3.pptx]

## Slide 1
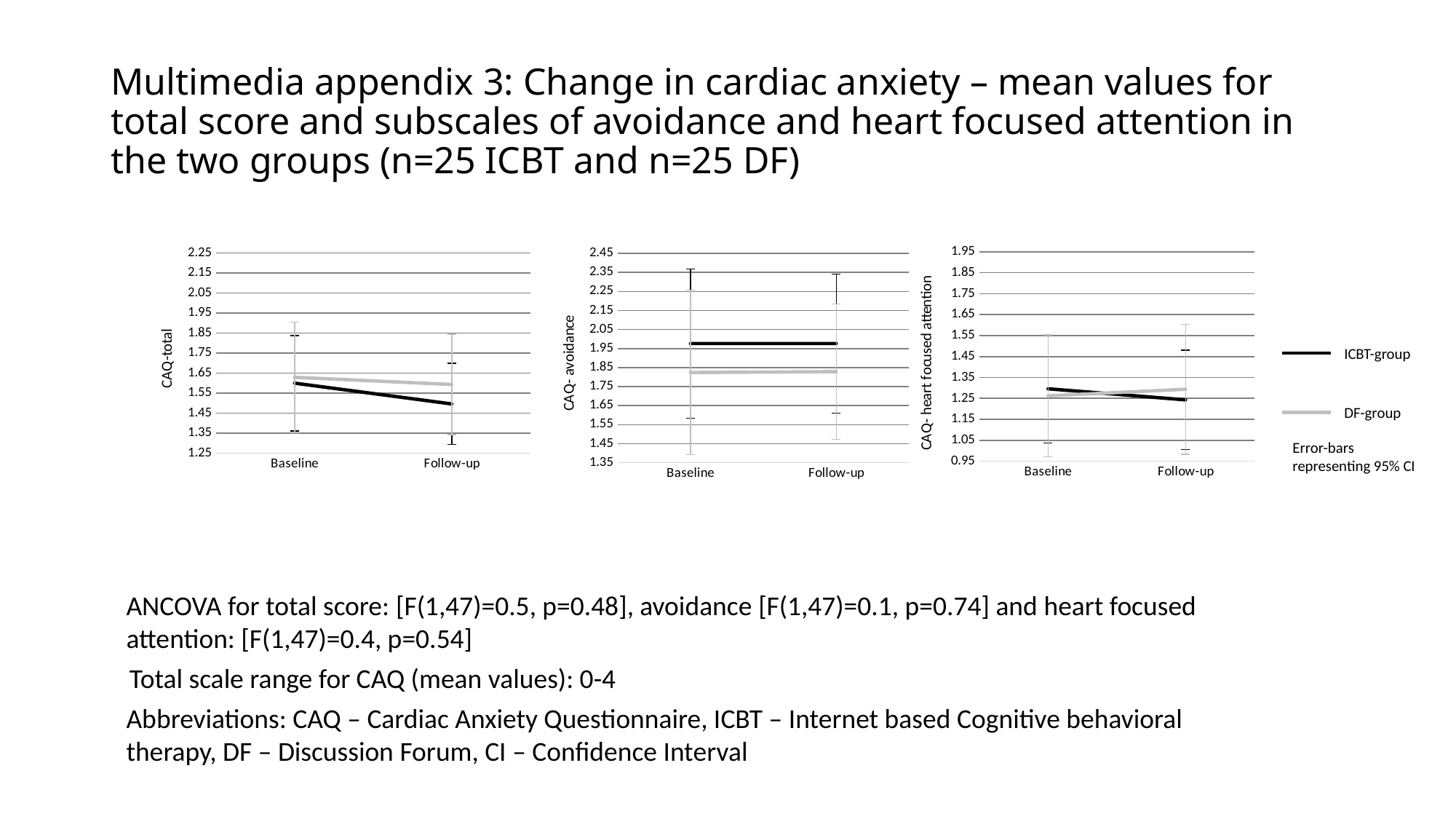

# Multimedia appendix 3: Change in cardiac anxiety – mean values for total score and subscales of avoidance and heart focused attention in the two groups (n=25 ICBT and n=25 DF)
### Chart
| Category | ICBT Heart focused attention | DF Heart focused attention |
|---|---|---|
| Baseline | 1.2960000000000003 | 1.262333333333333 |
| Follow-up | 1.242927 | 1.293659 |
### Chart
| Category | ICBT-group total | DF-group total |
|---|---|---|
| Baseline | 1.6 | 1.629166666666666 |
| Follow-up | 1.496043 | 1.593388 |
### Chart
| Category | ICBT Avoidance | DF Avoidance |
|---|---|---|
| Baseline | 1.976 | 1.824 |
| Follow-up | 1.97639 | 1.828488 |ICBT-group
DF-group
Error-bars representing 95% CI
ANCOVA for total score: [F(1,47)=0.5, p=0.48], avoidance [F(1,47)=0.1, p=0.74] and heart focused attention: [F(1,47)=0.4, p=0.54]
Total scale range for CAQ (mean values): 0-4
Abbreviations: CAQ – Cardiac Anxiety Questionnaire, ICBT – Internet based Cognitive behavioral therapy, DF – Discussion Forum, CI – Confidence Interval
