# Supplementary material for: The Effect of Guided Web-Based Cognitive Behavioral Therapy on Patients With Depressive Symptoms and Heart Failure: A Pilot Randomized Controlled Trial
Source: J Med Internet Res. 2016 Aug 3;18(8):e194. doi: 10.2196/jmir.5556 (PMC5070581; doi:10.2196/jmir.5556)
Supplement: Multimedia Appendix 4 [file jmir_v18i8e194_app4.pptx]

## Slide 1
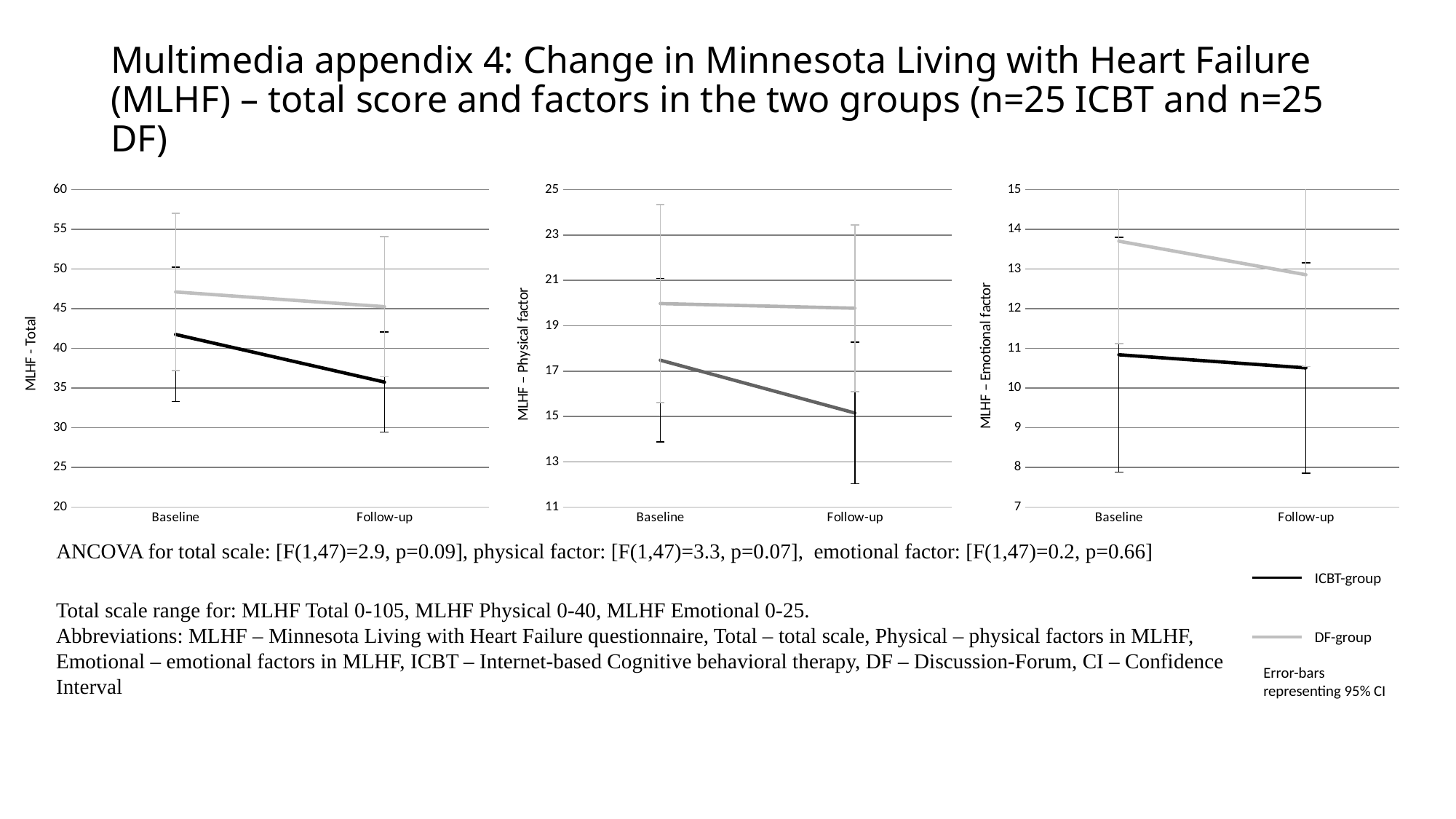

# Multimedia appendix 4: Change in Minnesota Living with Heart Failure (MLHF) – total score and factors in the two groups (n=25 ICBT and n=25 DF)
### Chart
| Category | ICBT-group total | DF-group total |
|---|---|---|
| Baseline | 41.76 | 47.115 |
| Follow-up | 35.762 | 45.26243902439024 |
### Chart
| Category | ICBT-group Phys. | DF-group Phys. |
|---|---|---|
| Baseline | 17.48 | 19.9783333333333 |
| Follow-up | 15.154146341463415 | 19.7726829268293 |
### Chart
| Category | ICBT-group Emo. | DF-group Emo. |
|---|---|---|
| Baseline | 10.84 | 13.701666666666668 |
| Follow-up | 10.5093 | 12.85658536585366 |ANCOVA for total scale: [F(1,47)=2.9, p=0.09], physical factor: [F(1,47)=3.3, p=0.07], emotional factor: [F(1,47)=0.2, p=0.66]
ICBT-group
DF-group
Error-bars representing 95% CI
Total scale range for: MLHF Total 0-105, MLHF Physical 0-40, MLHF Emotional 0-25.
Abbreviations: MLHF – Minnesota Living with Heart Failure questionnaire, Total – total scale, Physical – physical factors in MLHF, Emotional – emotional factors in MLHF, ICBT – Internet-based Cognitive behavioral therapy, DF – Discussion-Forum, CI – Confidence Interval
