# Supplementary material for: The Effect of Guided Web-Based Cognitive Behavioral Therapy on Patients With Depressive Symptoms and Heart Failure: A Pilot Randomized Controlled Trial
Source: J Med Internet Res. 2016 Aug 3;18(8):e194. doi: 10.2196/jmir.5556 (PMC5070581; doi:10.2196/jmir.5556)
Supplement: Multimedia Appendix 5 [file jmir_v18i8e194_app5.pdf]

# CONSORT-EHEALTH (V 1.6.1) - Submission/Publication Form

The CONSORT-EHEALTH checklist is intended for authors of randomized trials evaluating web-based and Internet-based applications/interventions, including mobile interventions, electronic games (incl multiplayer games), social media, certain telehealth applications, and other interactive and/or networked electronic applications. Some of the items (e.g. all subitems under item 5 - description of the intervention) may also be applicable for other study designs.

The goal of the CONSORT EHEALTH checklist and guideline is to be
a) a guide for reporting for authors of RCTs,
b) to form a basis for appraisal of an ehealth trial (in terms of validity)

CONSORT-EHEALTH items/subitems are MANDATORY reporting items for studies published in the Journal of Medical Internet Research and other journals / scientific societies endorsing the checklist.

Items numbered 1., 2., 3., 4a., 4b etc are original CONSORT or CONSORT-NPT (non-pharmacologic treatment) items.
Items with Roman numerals (i., ii, iii, iv etc.) are CONSORT-EHEALTH extensions/clarifications.

As the CONSORT-EHEALTH checklist is still considered in a formative stage, we would ask that you also RATE ON A SCALE OF 1-5 how important/useful you feel each item is FOR THE PURPOSE OF THE CHECKLIST and reporting guideline (optional).

Mandatory reporting items are marked with a red *.
In the textboxes, either copy & paste the relevant sections from your manuscript into this form - please include any quotes from your manuscript in QUOTATION MARKS, or answer directly by providing additional information not in the manuscript, or elaborating on why the item was not relevant for this study.

YOUR ANSWERS WILL BE PUBLISHED AS A SUPPLEMENTARY FILE TO YOUR PUBLICATION IN JMIR AND ARE CONSIDERED PART OF YOUR PUBLICATION (IF ACCEPTED).
Please fill in these questions diligently. Information will not be copyedited, so please use proper spelling and grammar, use correct capitalization, and avoid abbreviations.

DO NOT FORGET TO SAVE AS PDF _AND_ CLICK THE SUBMIT BUTTON SO YOUR ANSWERS ARE IN OUR DATABASE !!!

Citation Suggestion (if you append the pdf as Appendix we suggest to cite this paper in the caption):
Eysenbach G, CONSORT-EHEALTH Group
CONSORT-EHEALTH: Improving and Standardizing Evaluation Reports of Web-based and Mobile Health Interventions
J Med Internet Res 2011;13(4):e126
URL: http://www.jmir.org/2011/4/e126/
doi: 10.2196/jmir.1923
PMID: 22209829

*Obligatorisk

**Your name** *

First Last

Johan Lundgren

**Primary Affiliation (short), City, Country** *

University of Toronto, Toronto, Canada

Linköping University, Nor

**Your e-mail address** *

abc@gmail.com

johan.lundgren@liu.se

**Title of your manuscript** *

Provide the (draft) title of your manuscript.

The effect of guided web-based cognitive behavioral therapy on patients with depressive symptoms and heart failure- A randomized controlled trial

**Article Preparation Status/Stage** *

At which stage in your article preparation are you currently (at the time you fill in this form)

- ○ not submitted yet - in early draft status
- ◉ not submitted yet - in late draft status, just before submission
- ○ submitted to a journal but not reviewed yet
- ○ submitted to a journal and after receiving initial reviewer comments
- ○ submitted to a journal and accepted, but not published yet
- ○ published
- ○ Övrigt:

**Journal** *

If you already know where you will submit this paper (or if it is already submitted), please provide the journal name (if it is not JMIR, provide the journal name under "other")

- ○ not submitted yet / unclear where I will submit this
- ◉ Journal of Medical Internet Research (JMIR)
- ○ Övrigt:

**Manuscript tracking number** *

If this is a JMIR submission, please provide the manuscript tracking number under "other" (The ms tracking number can be found in the submission acknowledgement email, or when you login as author in JMIR. If the paper is already published in JMIR, then the ms tracking number is the four-digit number at the end of the DOI, to be found at the bottom of each published article in JMIR)

- ◉ no ms number (yet) / not (yet) submitted to / published in JMIR

○ Övrigt: [ ]

# TITLE AND ABSTRACT

## 1a) TITLE: Identification as a randomized trial in the title

**1a) Does your paper address CONSORT item 1a? ***

I.e does the title contain the phrase "Randomized Controlled Trial"? (if not, explain the reason under "other")

◉ yes

○ Övrigt: [ ]

**1a-i) Identify the mode of delivery in the title**

Identify the mode of delivery. Preferably use "web-based" and/or "mobile" and/or "electronic game" in the title. Avoid ambiguous terms like "online", "virtual", "interactive". Use "Internet-based" only if Intervention includes non-web-based Internet components (e.g. email), use "computer-based" or "electronic" only if offline products are used. Use "virtual" only in the context of "virtual reality" (3-D worlds). Use "online" only in the context of "online support groups". Complement or substitute product names with broader terms for the class of products (such as "mobile" or "smart phone" instead of "iphone"), especially if the application runs on different platforms.

| | 1 | 2 | 3 | 4 | 5 | |
|---|---|---|---|---|---|---|
| subitem not at all important | ○ | ○ | ◉ | ○ | ○ | essential |

**Does your paper address subitem 1a-i? ***

Copy and paste relevant sections from manuscript title (include quotes in quotation marks "like this" to indicate direct quotes from your manuscript), or elaborate on this item by providing additional information not in the ms, or briefly explain why the item is not applicable/relevant for your study

"effect of guided web-based cognitive behavioral therapy"

**1a-ii) Non-web-based components or important co-interventions in title**

Mention non-web-based components or important co-interventions in title, if any (e.g., "with telephone support").

| | 1 | 2 | 3 | 4 | 5 | |
|---|---|---|---|---|---|---|
| subitem not at all important | ○ | ○ | ○ | ◉ | ○ | essential |

**Does your paper address subitem 1a-ii?**

Copy and paste relevant sections from manuscript title (include quotes in quotation marks "like this" to indicate direct quotes from your manuscript), or elaborate on this item by providing additional information not in the ms, or briefly explain why the item is not applicable/relevant for your study

N/A, intervention (and control group) did not include any non-web-based or non-internet-based components. Pre-intervention contact during the inclusion process, to assess potential participants and prevent multiple registrations by telephone clearly described in methods section " Candidates were contacted by telephone to check any uncertainties in the screening forms, and to prevent multiple registrations"

**1a-iii) Primary condition or target group in the title**

Mention primary condition or target group in the title, if any (e.g., "for children with Type I Diabetes") Example: A Web-based and Mobile Intervention with Telephone Support for Children with Type I Diabetes: Randomized Controlled Trial

| | 1 | 2 | 3 | 4 | 5 | |
|---|---|---|---|---|---|---|
| subitem not at all important | ○ | ○ | ○ | ◉ | ○ | essential |

**Does your paper address subitem 1a-iii? ***

Copy and paste relevant sections from manuscript title (include quotes in quotation marks "like this" to indicate direct quotes from your manuscript), or elaborate on this item by providing additional information not in the ms, or briefly explain why the item is not applicable/relevant for your study

"on patients with depressive symptoms and heart failure"

# 1b) ABSTRACT: Structured summary of trial design, methods, results, and conclusions

NPT extension: Description of experimental treatment, comparator, care providers, centers, and blinding status.

**1b-i) Key features/functionalities/components of the intervention and comparator in the METHODS section of the ABSTRACT**

Mention key features/functionalities/components of the intervention and comparator in the abstract. If possible, also mention theories and principles used for designing the site. Keep in mind the needs of systematic reviewers and indexers by including important synonyms. (Note: Only report in the abstract what the main paper is reporting. If this information is missing from the main body of text, consider adding it)

| | 1 | 2 | 3 | 4 | 5 | |
|---|---|---|---|---|---|---|
| subitem not at all important | ○ | ○ | ○ | ◉ | ○ | essential |

**Does your paper address subitem 1b-i? ***

Copy and paste relevant sections from the manuscript abstract (include quotes in quotation marks "like this" to indicate direct quotes from your manuscript), or elaborate on this item by providing additional information not in the ms, or briefly explain why the item is not applicable/relevant for your study

"Internet-based cognitive behavioral therapy (CBT) as guided self-help CBT programs (ICBT) have shown good effects in the treatment of depression. Until now ICBT has not previously been evaluated in patients with HF with depressive symptoms."

"The aims of this study were to: (I) evaluate the effect of a nine-week guided ICBT program on depressive symptoms in patients with HF"

**1b-ii) Level of human involvement in the METHODS section of the ABSTRACT**

Clarify the level of human involvement in the abstract, e.g., use phrases like "fully automated" vs. "therapist/nurse/care provider/physician-assisted" (mention number and expertise of providers involved, if any). (Note: Only report in the abstract what the main paper is reporting. If this information is missing from the main body of text, consider adding it)

| | 1 | 2 | 3 | 4 | 5 | |
|---|---|---|---|---|---|---|
| subitem not at all important | ○ | ○ | ◉ | ○ | ○ | essential |

**Does your paper address subitem 1b-ii?**

Copy and paste relevant sections from the manuscript abstract (include quotes in quotation marks "like this" to indicate direct quotes from your manuscript), or elaborate on this item by providing additional information not in the ms, or briefly explain why the item is not applicable/relevant for your study

"evaluate the effect of a nine-week guided ICBT program"

**1b-iii) Open vs. closed, web-based (self-assessment) vs. face-to-face assessments in the METHODS section of the ABSTRACT**

Mention how participants were recruited (online vs. offline), e.g., from an open access website or from a clinic or a closed online user group (closed usergroup trial), and clarify if this was a purely web-based trial, or there were face-to-face components (as part of the intervention or for assessment). Clearly say if outcomes were self-assessed through questionnaires (as common in web-based trials). Note: In traditional offline trials, an open trial (open-label trial) is a type of clinical trial in which both the researchers and participants know which treatment is being administered. To avoid confusion, use "blinded" or "unblinded" to indicated the level of blinding instead of "open", as "open" in web-based trials usually refers to "open access" (i.e. participants can self-enrol). (Note: Only report in the abstract what the main paper is reporting. If this information is missing from the main body of text, consider adding it)

| | 1 | 2 | 3 | 4 | 5 | |
|---|---|---|---|---|---|---|
| subitem not at all important | ○ | ◉ | ○ | ○ | ○ | essential |

**Does your paper address subitem 1b-iii?**

Copy and paste relevant sections from the manuscript abstract (include quotes in quotation marks "like this" to indicate direct quotes from your manuscript), or elaborate on this item by providing additional information not in the ms, or briefly explain why the item is not applicable/relevant for your study

". Data (self-reported) were collected at baseline and at the nine-week follow-up."

### 1b-iv) RESULTS section in abstract must contain use data

Report number of participants enrolled/assessed in each group, the use/uptake of the intervention (e.g., attrition/adherence metrics, use over time, number of logins etc.), in addition to primary/secondary outcomes. (Note: Only report in the abstract what the main paper is reporting. If this information is missing from the main body of text, consider adding it)

| | 1 | 2 | 3 | 4 | 5 | |
|---|---|---|---|---|---|---|
| subitem not at all important | ○ | ◉ | ○ | ○ | ○ | essential |

### Does your paper address subitem 1b-iv?

Copy and paste relevant sections from the manuscript abstract (include quotes in quotation marks "like this" to indicate direct quotes from your manuscript), or elaborate on this item by providing additional information not in the ms, or briefly explain why the item is not applicable/relevant for your study

Number of participants in each group stated in the result section in the abstract: "In the ICBT group (n=25)" and "In the DF group (n=25) "
Attrition/adherence metrics left out in abstract due to space limitation, but is reported in the manuscripts result section.

### 1b-v) CONCLUSIONS/DISCUSSION in abstract for negative trials

Conclusions/Discussions in abstract for negative trials: Discuss the primary outcome - if the trial is negative (primary outcome not changed), and the intervention was not used, discuss whether negative results are attributable to lack of uptake and discuss reasons. (Note: Only report in the abstract what the main paper is reporting. If this information is missing from the main body of text, consider adding it)

| | 1 | 2 | 3 | 4 | 5 | |
|---|---|---|---|---|---|---|
| subitem not at all important | ○ | ○ | ◉ | ○ | ○ | essential |

### Does your paper address subitem 1b-v?

Copy and paste relevant sections from the manuscript abstract (include quotes in quotation marks "like this" to indicate direct quotes from your manuscript), or elaborate on this item by providing additional information not in the ms, or briefly explain why the item is not applicable/relevant for your study

In the abstract this is not clearly discussed, partly due to space limitation, partly due to that author instruction for JMIR does not include discussion in the structure of the abstract.
However, the conclusion formulated so that it is clear that we did not find any statistically significant differences between the intervention group (ICBT) and the control group (receiving a online moderated discussion forum) "Guided ICBT adapted for persons with HF and depressive symptoms can potentially

# INTRODUCTION

## 2a) In INTRODUCTION: Scientific background and explanation of rationale

### 2a-i) Problem and the type of system/solution

Describe the problem and the type of system/solution that is object of the study: intended as stand-alone intervention vs. incorporated in broader health care program? Intended for a particular patient population? Goals of the intervention, e.g., being more cost-effective to other interventions, replace or complement other solutions? (Note: Details about the intervention are provided in "Methods" under 5)

| | 1 | 2 | 3 | 4 | 5 | |
|---|---|---|---|---|---|---|
| subitem not at all important | ○ | ○ | ○ | ◉ | ○ | essential |

**Does your paper address subitem 2a-i? ***

Copy and paste relevant sections from the manuscript (include quotes in quotation marks "like this" to indicate direct quotes from your manuscript), or elaborate on this item by providing additional information not in the ms, or briefly explain why the item is not applicable/relevant for your study

"Depressive symptoms are common in patients with heart failure (HF) (20-40%)[1-3]. They lead to higher morbidity and mortality and diminish self-care and health-related quality of life (QoL) [3]. However, treatment strategies for depressive symptoms in patients with HF still remain to be established [3, 4]."
". ICBT has been shown to be a good and time-efficient method for the treatment of depressive symptoms, and also

### 2a-ii) Scientific background, rationale: What is known about the (type of) system

Scientific background, rationale: What is known about the (type of) system that is the object of the study (be sure to discuss the use of similar systems for other conditions/diagnoses, if appropiate), motivation for the study, i.e. what are the reasons for and what is the context for this specific study, from which stakeholder viewpoint is the study performed, potential impact of findings [2]. Briefly justify the choice of the comparator.

| | 1 | 2 | 3 | 4 | 5 | |
|---|---|---|---|---|---|---|
| subitem not at all important | ○ | ○ | ◉ | ○ | ○ | essential |

**Does your paper address subitem 2a-ii? ***

Copy and paste relevant sections from the manuscript (include quotes in quotation marks "like this" to indicate direct quotes from your manuscript), or elaborate on this item by providing additional information not in the ms, or briefly explain why the item is not applicable/relevant for your study

"In general depressive symptoms can be treated, by psychotherapy or by pharmacology[12]. However, the impact of pharmacological treatment of depression in HF is not clear [4] and may be complicated due to an already complex pharmacological treatment regime [2]. Furthermore, patients with heart disease seem to prefer talking therapies such as cognitive behavioral therapy (CBT) over pharmacological treatment [8].
[illegible]

## 2b) In INTRODUCTION: Specific objectives or hypotheses

**Does your paper address CONSORT subitem 2b? ***

Copy and paste relevant sections from the manuscript (include quotes in quotation marks "like this" to indicate direct quotes from your manuscript), or elaborate on this item by providing additional information not in the ms, or briefly explain why the item is not applicable/relevant for your study

"The primary aim of this study was therefore to evaluate the effect of ICBT on depressive symptoms in patients with HF. Since the frequency of participation in the CBT treatment [18], level of depressive symptoms pre-intervention [19], age [20], sex and New York Heart Association Class (NYHA class)[2] may impact changes in depressive symptoms, a second aim was to assess these factors' associations with the change in depressive symptoms. A third aim was to investigate the effect [illegible]

# METHODS

## 3a) Description of trial design (such as parallel, factorial) including allocation ratio

**Does your paper address CONSORT subitem 3a? ***

Copy and paste relevant sections from the manuscript (include quotes in quotation marks "like this" to indicate direct quotes from your manuscript), or elaborate on this item by providing additional information not in the ms, or briefly explain why the item is not applicable/relevant for your study

"An open label, randomized control design was used."
"Randomization was performed by a person blinded to screening and baseline data using Stata v.13 proc Ralloc with a block size of two."

# 3b) Important changes to methods after trial commencement (such as eligibility criteria), with reasons

**Does your paper address CONSORT subitem 3b? ***

Copy and paste relevant sections from the manuscript (include quotes in quotation marks "like this" to indicate direct quotes from your manuscript), or elaborate on this item by providing additional information not in the ms, or briefly explain why the item is not applicable/relevant for your study

N/A, no such changes made after commencement of this study.

**3b-i) Bug fixes, Downtimes, Content Changes**

Bug fixes, Downtimes, Content Changes: ehealth systems are often dynamic systems. A description of changes to methods therefore also includes important changes made on the intervention or comparator during the trial (e.g., major bug fixes or changes in the functionality or content) (5-iii) and other "unexpected events" that may have influenced study design such as staff changes, system failures/downtimes, etc. [2].

| | 1 | 2 | 3 | 4 | 5 | |
|---|---|---|---|---|---|---|
| subitem not at all important | ○ | ○ | ○ | ◉ | ○ | essential |

**Does your paper address subitem 3b-i?**

Copy and paste relevant sections from the manuscript (include quotes in quotation marks "like this" to indicate direct quotes from your manuscript), or elaborate on this item by providing additional information not in the ms, or briefly explain why the item is not applicable/relevant for your study

No such actions needed during the study period. However changes made in the content after the proof of concept study performed previously is reported: "Compared to the ICBT-program tested in the proof of concept study, a short non-mandatory relaxation exercise was added in module three. No changes were made to the program during the trial."

# 4a) Eligibility criteria for participants

**Does your paper address CONSORT subitem 4a? ***

Copy and paste relevant sections from the manuscript (include quotes in quotation marks "like this" to indicate direct quotes from your manuscript), or elaborate on this item by providing additional information not in the ms, or briefly explain why the item is not applicable/relevant for your study

"To recruit participants an information letter was sent to all patients who had an outpatient appointment or who had been admitted to hospital with the main diagnosis of HF during 2013 and 2014 in four hospitals in the south-east of Sweden..." "Inclusion criteria were: at least mild depressive symptoms (PHQ-9 ≥5), regular access to a computer with an internet connection, access to a cellphone, being motivated to participate in treatment of depressive symptoms, and being [illegible]

### 4a-i) Computer / Internet literacy

Computer / Internet literacy is often an implicit "de facto" eligibility criterion - this should be explicitly clarified.

| | 1 | 2 | 3 | 4 | 5 | |
|---|---|---|---|---|---|---|
| subitem not at all important | ○ | ○ | ○ | ◉ | ○ | essential |

### Does your paper address subitem 4a-i?

Copy and paste relevant sections from the manuscript (include quotes in quotation marks "like this" to indicate direct quotes from your manuscript), or elaborate on this item by providing additional information not in the ms, or briefly explain why the item is not applicable/relevant for your study

"Computer / Internet literacy was not a criterion for inclusion or exclusion. However, recipients interested in participation had to register at the study web-site and completed an online screening form..."

### 4a-ii) Open vs. closed, web-based vs. face-to-face assessments:

Open vs. closed, web-based vs. face-to-face assessments: Mention how participants were recruited (online vs. offline), e.g., from an open access website or from a clinic, and clarify if this was a purely web-based trial, or there were face-to-face components (as part of the intervention or for assessment), i.e., to what degree got the study team to know the participant. In online-only trials, clarify if participants were quasi-anonymous and whether having multiple identities was possible or whether technical or logistical measures (e.g., cookies, email confirmation, phone calls) were used to detect/prevent these.

| | 1 | 2 | 3 | 4 | 5 | |
|---|---|---|---|---|---|---|
| subitem not at all important | ○ | ○ | ○ | ◉ | ○ | essential |

### Does your paper address subitem 4a-ii? *

Copy and paste relevant sections from the manuscript (include quotes in quotation marks "like this" to indicate direct quotes from your manuscript), or elaborate on this item by providing additional information not in the ms, or briefly explain why the item is not applicable/relevant for your study

To find possible candidate for the intervention hospital records over patients treated for heart failure were used and a letter with information about the study was sent home to these patients (previously tested recruitment by advertisement in news-papers proved to be ineffective in our proof of concept study). "To recruit participants an information letter was sent to all patients who had an outpatient appointment or who had been admitted to hospital with the main diagnosis of HF during [illegible]

### 4a-iii) Information giving during recruitment

Information given during recruitment. Specify how participants were briefed for recruitment and in the informed consent procedures (e.g., publish the informed consent documentation as appendix, see also item X26), as this information may have an effect on user self-selection, user expectation and may also bias results.

| | 1 | 2 | 3 | 4 | 5 | |
|---|---|---|---|---|---|---|
| subitem not at all important | ○ | ○ | ◉ | ○ | ○ | essential |

### Does your paper address subitem 4a-iii?

Copy and paste relevant sections from the manuscript (include quotes in quotation marks "like this" to indicate direct quotes from your manuscript), or elaborate on this item by providing additional information not in the ms, or briefly explain why the item is not applicable/relevant for your study

"To recruit participants an information letter was sent to all patients who had an outpatient appointment or who had been admitted to hospital with the main diagnosis of HF during 2013 and 2014 in four hospitals in the south-east of Sweden"
The information described that the study had the aim of investigating the effect of the ICBT in patients with HF and stated the inclusion criteria for the study. Patients that had felt them self depressed or down hearted during at least two [illegible]

# 4b) Settings and locations where the data were collected

### Does your paper address CONSORT subitem 4b? *

Copy and paste relevant sections from the manuscript (include quotes in quotation marks "like this" to indicate direct quotes from your manuscript), or elaborate on this item by providing additional information not in the ms, or briefly explain why the item is not applicable/relevant for your study

"However, recipients interested in participation had to register at the study web-site and completed an online screening form including the assessment of depressive symptoms by means of the Patient Health Questionnaire –9 (PHQ-9), self-reported use of medication, co-morbidities, NYHA class and demographics."

"Self-assessed data were collected online at baseline (before [illegible]

### 4b-i) Report if outcomes were (self-)assessed through online questionnaires

Clearly report if outcomes were (self-)assessed through online questionnaires (as common in web-based trials) or otherwise.

1 2 3 4 5

subitem not at all important ○ ○ ◉ ○ ○ essential

**Does your paper address subitem 4b-i? ***

Copy and paste relevant sections from the manuscript (include quotes in quotation marks "like this" to indicate direct quotes from your manuscript), or elaborate on this item by providing additional information not in the ms, or briefly explain why the item is not applicable/relevant for your study

"Self-assessed data were collected online at baseline (before the start of the intervention) and after the end of week nine in the intervention."

**4b-ii) Report how institutional affiliations are displayed**

Report how institutional affiliations are displayed to potential participants [on ehealth media], as affiliations with prestigious hospitals or universities may affect volunteer rates, use, and reactions with regards to an intervention.(Not a required item – describe only if this may bias results)

| | 1 | 2 | 3 | 4 | 5 | |
|---|---|---|---|---|---|---|
| subitem not at all important | ○ | ◉ | ○ | ○ | ○ | essential |

**Does your paper address subitem 4b-ii?**

Copy and paste relevant sections from the manuscript (include quotes in quotation marks "like this" to indicate direct quotes from your manuscript), or elaborate on this item by providing additional information not in the ms, or briefly explain why the item is not applicable/relevant for your study

This is not clearly addressed in the manuscript. However in the information letter sent out it is stated that the study is performed in a cooperation between the university and a university hospital in south east Sweden. On the study web-site part of the research team is presented and affiliation to there university is possible to find, but no logotypes for the university or the university hospital is used on the web-site, nor is the URL of the site associated with any of them.

# 5) The interventions for each group with sufficient details to allow replication, including how and when they were actually administered

**5-i) Mention names, credential, affiliations of the developers, sponsors, and owners**

Mention names, credential, affiliations of the developers, sponsors, and owners [6] (if authors/evaluators are owners or developer of the software, this needs to be declared in a "Conflict of interest" section or mentioned elsewhere in the manuscript).

| | 1 | 2 | 3 | 4 | 5 | |
|---|---|---|---|---|---|---|
| subitem not at all important | | | | | | essential |

○ ○ ◉ ○ ○

**Does your paper address subitem 5-i?**

Copy and paste relevant sections from the manuscript (include quotes in quotation marks "like this" to indicate direct quotes from your manuscript), or elaborate on this item by providing additional information not in the ms, or briefly explain why the item is not applicable/relevant for your study

"Intervention procedure
Each participant received a password and an unidentifiable username. Login to the web-portal (to access the treatment program, feedback, secure e-mail, and assessment forms) required a two-factor authentication system to protect sensitive information. If technical problems occurred, both the therapist and the participants could get support from a computer technician.
[illegible]

**5-ii) Describe the history/development process**

Describe the history/development process of the application and previous formative evaluations (e.g., focus groups, usability testing), as these will have an impact on adoption/use rates and help with interpreting results.

| | 1 | 2 | 3 | 4 | 5 | |
|---|---|---|---|---|---|---|
| subitem not at all important | ○ | ○ | ○ | ◉ | ○ | essential |

**Does your paper address subitem 5-ii?**

Copy and paste relevant sections from the manuscript (include quotes in quotation marks "like this" to indicate direct quotes from your manuscript), or elaborate on this item by providing additional information not in the ms, or briefly explain why the item is not applicable/relevant for your study

The development process of the intervention is described in in short with focus on the procedure. The development process has been described in detail in a previously published manuscript (reference given in text in this manuscript) and changes compared to the intervention in the proof of concept study is clearly stated: "Each participant received a password and an unidentifiable username. Login to the web-portal (to access the treatment program, feedback, secure e-mail, and
[illegible]

**5-iii) Revisions and updating**

Revisions and updating. Clearly mention the date and/or version number of the application/intervention (and comparator, if applicable) evaluated, or describe whether the intervention underwent major changes during the evaluation process, or whether the development and/or content was "frozen" during the trial. Describe dynamic components such as news feeds or changing content which may have an impact on the replicability of the intervention (for unexpected events see item 3b).

| | 1 | 2 | 3 | 4 | 5 | |
|---|---|---|---|---|---|---|
| subitem not at all important | ○ | ○ | ○ | ◉ | ○ | essential |

**Does your paper address subitem 5-iii?**

Copy and paste relevant sections from the manuscript (include quotes in quotation marks "like this" to indicate direct quotes from your manuscript), or elaborate on this item by providing

additional information not in the ms, or briefly explain why the item is not applicable/relevant for your study

"Compared to the ICBT-program tested in the proof of concept study, a short non-mandatory relaxation exercise was added in module three. No changes were made to the program during the trial."

### 5-iv) Quality assurance methods

Provide information on quality assurance methods to ensure accuracy and quality of information provided [1], if applicable.

| | 1 | 2 | 3 | 4 | 5 | |
|---|---|---|---|---|---|---|
| subitem not at all important | ○ | ○ | ◉ | ○ | ○ | essential |

### Does your paper address subitem 5-iv?

Copy and paste relevant sections from the manuscript (include quotes in quotation marks "like this" to indicate direct quotes from your manuscript), or elaborate on this item by providing additional information not in the ms, or briefly explain why the item is not applicable/relevant for your study

"Each participant received a password and an unidentifiable username. Login to the web-portal (to access the treatment program, feedback, secure e-mail, and assessment forms) required a two-factor authentication system to protect sensitive information. If technical problems occurred, both the therapist and the participants could get support from a computer technician."

### 5-v) Ensure replicability by publishing the source code, and/or providing screenshots/screen-capture video, and/or providing flowcharts of the algorithms used

Ensure replicability by publishing the source code, and/or providing screenshots/screen-capture video, and/or providing flowcharts of the algorithms used. Replicability (i.e., other researchers should in principle be able to replicate the study) is a hallmark of scientific reporting.

| | 1 | 2 | 3 | 4 | 5 | |
|---|---|---|---|---|---|---|
| subitem not at all important | ○ | ○ | ○ | ◉ | ○ | essential |

### Does your paper address subitem 5-v?

Copy and paste relevant sections from the manuscript (include quotes in quotation marks "like this" to indicate direct quotes from your manuscript), or elaborate on this item by providing additional information not in the ms, or briefly explain why the item is not applicable/relevant for your study

Flowchart of participants presented in figure 1. Screen shots of part of the web-based platform presented in figure 2. A multimedia appendix presents a more comprehensive description of the platform. Table 1 gives an overview of the ICBT-program and the discussion forum.

"To summarize, the ICBT program consisted of seven consecutive modules that were worked with over nine weeks

### 5-vi) Digital preservation

Digital preservation: Provide the URL of the application, but as the intervention is likely to change or disappear over the course of the years; also make sure the intervention is archived (Internet Archive, webcitation.org, and/or publishing the source code or screenshots/videos alongside the article). As pages behind login screens cannot be archived, consider creating demo pages which are accessible without login.

| | 1 | 2 | 3 | 4 | 5 | |
|---|---|---|---|---|---|---|
| subitem not at all important | ○ | ◉ | ○ | ○ | ○ | essential |

### Does your paper address subitem 5-vi?

Copy and paste relevant sections from the manuscript (include quotes in quotation marks "like this" to indicate direct quotes from your manuscript), or elaborate on this item by providing additional information not in the ms, or briefly explain why the item is not applicable/relevant for your study

Archiving by Internet Archives not possible due to technical reasons. Multimedia appendix contains videos on the pages behind login screens.

### 5-vii) Access

Access: Describe how participants accessed the application, in what setting/context, if they had to pay (or were paid) or not, whether they had to be a member of specific group. If known, describe how participants obtained "access to the platform and Internet" [1]. To ensure access for editors/reviewers/readers, consider to provide a "backdoor" login account or demo mode for reviewers/readers to explore the application (also important for archiving purposes, see vi).

| | 1 | 2 | 3 | 4 | 5 | |
|---|---|---|---|---|---|---|
| subitem not at all important | ○ | ○ | ◉ | ○ | ○ | essential |

### Does your paper address subitem 5-vii? *

Copy and paste relevant sections from the manuscript (include quotes in quotation marks "like this" to indicate direct quotes from your manuscript), or elaborate on this item by providing additional information not in the ms, or briefly explain why the item is not applicable/relevant for your study

"No economical compensation was given to the participants"
Participants had to provide there own internet access during the study. Access to the platform was obtained after inclusion in the study and by to factor identification. How participants accessed the Internet is not investigated or recorded in this study.

### 5-viii) Mode of delivery, features/functionalities/components of the intervention and comparator, and the theoretical framework

Describe mode of delivery, features/functionalities/components of the intervention and comparator, and the theoretical framework [6] used to design them (instructional strategy [1], behaviour change techniques, persuasive features, etc., see e.g., [7, 8] for terminology). This includes an in-depth description of the content (including where it is coming from and who developed it) [1]," whether [and how] it is tailored to individual circumstances and allows users to track their progress and receive feedback" [6]. This also includes a description of communication delivery channels and – if computer-mediated communication is a component – whether communication was synchronous or asynchronous [6]. It also includes information on presentation strategies [1], including page design principles, average amount of text on pages, presence of hyperlinks to other resources, etc. [1].

| | 1 | 2 | 3 | 4 | 5 | |
|---|---|---|---|---|---|---|
| subitem not at all important | ○ | ○ | ◉ | ○ | ○ | essential |

### Does your paper address subitem 5-viii? *

Copy and paste relevant sections from the manuscript (include quotes in quotation marks "like this" to indicate direct quotes from your manuscript), or elaborate on this item by providing additional information not in the ms, or briefly explain why the item is not applicable/relevant for your study

Some of this has been described in the previously published proof of concept study where the theoretical underpinning for the intervention is given. (reference given in the manuscript)

"Each module contained texts to be read and assignments to be completed by the participants. Written feedback was provided on each assignment. Participants could ask questions about the feedback or the content of the module using the [illegible]

### 5-ix) Describe use parameters

Describe use parameters (e.g., intended "doses" and optimal timing for use). Clarify what instructions or recommendations were given to the user, e.g., regarding timing, frequency, heaviness of use, if any, or was the intervention used ad libitum.

| | 1 | 2 | 3 | 4 | 5 | |
|---|---|---|---|---|---|---|
| subitem not at all important | ○ | ○ | ◉ | ○ | ○ | essential |

### Does your paper address subitem 5-ix?

Copy and paste relevant sections from the manuscript (include quotes in quotation marks "like this" to indicate direct quotes from your manuscript), or elaborate on this item by providing additional information not in the ms, or briefly explain why the item is not applicable/relevant for your study

"...the ICBT program consisted of seven consecutive modules that were worked with over nine weeks..."

**5-x) Clarify the level of human involvement**

Clarify the level of human involvement (care providers or health professionals, also technical assistance) in the e-intervention or as co-intervention (detail number and expertise of professionals involved, if any, as well as "type of assistance offered, the timing and frequency of the support, how it is initiated, and the medium by which the assistance is delivered". It may be necessary to distinguish between the level of human involvement required for the trial, and the level of human involvement required for a routine application outside of a RCT setting (discuss under item 21 – generalizability).

| | 1 | 2 | 3 | 4 | 5 | |
|---|---|---|---|---|---|---|
| subitem not at all important | ○ | ○ | ○ | ○ | ○ | essential |

**Does your paper address subitem 5-x?**

Copy and paste relevant sections from the manuscript (include quotes in quotation marks "like this" to indicate direct quotes from your manuscript), or elaborate on this item by providing additional information not in the ms, or briefly explain why the item is not applicable/relevant for your study

"Written feedback was provided on each assignment. Participants could ask questions about the feedback or the content of the module using the secure e-mail system. A mental health specialist nurse with experience of HF care provided feedback and answers within 24 hours. The mental health nurse was supervised by a clinical psychologist and could consult a cardiologist and nurses specialized in HF care if needed." ". If technical problems occurred, both the therapist [illegible]

**5-xi) Report any prompts/reminders used**

Report any prompts/reminders used: Clarify if there were prompts (letters, emails, phone calls, SMS) to use the application, what triggered them, frequency etc. It may be necessary to distinguish between the level of prompts/reminders required for the trial, and the level of prompts/reminders for a routine application outside of a RCT setting (discuss under item 21 – generalizability).

| | 1 | 2 | 3 | 4 | 5 | |
|---|---|---|---|---|---|---|
| subitem not at all important | ○ | ○ | ◉ | ○ | ○ | essential |

**Does your paper address subitem 5-xi? ***

Copy and paste relevant sections from the manuscript (include quotes in quotation marks "like this" to indicate direct quotes from your manuscript), or elaborate on this item by providing additional information not in the ms, or briefly explain why the item is not applicable/relevant for your study

"Participants who did not complete modules were reminded by e-mail."

**5-xii) Describe any co-interventions (incl. training/support)**

Describe any co-interventions (incl. training/support): Clearly state any interventions that are provided in addition to the targeted eHealth intervention, as ehealth intervention may not be designed as stand-alone intervention. This includes training sessions and support [1]. It may be necessary to distinguish between the level of training required for the trial, and the level of training for a routine application outside of a RCT setting (discuss under item 21 – generalizability.

| | 1 | 2 | 3 | 4 | 5 | |
|---|---|---|---|---|---|---|
| subitem not at all important | ○ | ○ | ◉ | ○ | ○ | essential |

**Does your paper address subitem 5-xii? ***

Copy and paste relevant sections from the manuscript (include quotes in quotation marks "like this" to indicate direct quotes from your manuscript), or elaborate on this item by providing additional information not in the ms, or briefly explain why the item is not applicable/relevant for your study

N/A

# 6a) Completely defined pre-specified primary and secondary outcome measures, including how and when they were assessed

**Does your paper address CONSORT subitem 6a? ***

Copy and paste relevant sections from the manuscript (include quotes in quotation marks "like this" to indicate direct quotes from your manuscript), or elaborate on this item by providing additional information not in the ms, or briefly explain why the item is not applicable/relevant for your study

Primary outcome: "Depressive symptoms were measured with the self-administered PHQ-9"
Secondary outcomes: "The Cardiac Anxiety Questionnaire (CAQ) [26] was used to measure cardiac-related anxiety." QoL was measured with the disease-specific instrument Minnesota Living with Heart Failure questionnaire (MLHF)" "Self-assessed data were collected online at baseline (before the start of the intervention) and after the end of week nine in the intervention."

**6a-i) Online questionnaires: describe if they were validated for online use and apply CHERRIES items to describe how the questionnaires were designed/deployed**

If outcomes were obtained through online questionnaires, describe if they were validated for online use and apply CHERRIES items to describe how the questionnaires were designed/deployed [9].

| | 1 | 2 | 3 | 4 | 5 | |
|---|---|---|---|---|---|---|
| subitem not at all important | ○ | ○ | ◉ | ○ | ○ | essential |

**Does your paper address subitem 6a-i?**

Copy and paste relevant sections from manuscript text

"The online version of PHQ-9 has demonstrated good inter-format reliability[25]"

**6a-ii) Describe whether and how "use" (including intensity of use/dosage) was defined/measured/monitored**

Describe whether and how "use" (including intensity of use/dosage) was defined/measured/monitored (logins, logfile analysis, etc.). Use/adoption metrics are important process outcomes that should be reported in any ehealth trial.

| | 1 | 2 | 3 | 4 | 5 | |
|---|---|---|---|---|---|---|
| subitem not at all important | ○ | ○ | ◉ | ○ | ○ | essential |

**Does your paper address subitem 6a-ii?**

Copy and paste relevant sections from manuscript text

"Activity in the program was calculated by the number of modules that the participants worked with (ICBT group only) as well as the number of logins to the web-portal during the nine-week period (both groups)."

**6a-iii) Describe whether, how, and when qualitative feedback from participants was obtained**

Describe whether, how, and when qualitative feedback from participants was obtained (e.g., through emails, feedback forms, interviews, focus groups).

| | 1 | 2 | 3 | 4 | 5 | |
|---|---|---|---|---|---|---|
| subitem not at all important | ○ | ○ | ○ | ◉ | ○ | essential |

**Does your paper address subitem 6a-iii?**

Copy and paste relevant sections from manuscript text

N/A (planned as separate study)

## 6b) Any changes to trial outcomes after the trial commenced, with reasons

**Does your paper address CONSORT subitem 6b? ***

Copy and paste relevant sections from the manuscript (include quotes in quotation marks "like this" to indicate direct quotes from your manuscript), or elaborate on this item by providing additional information not in the ms, or briefly explain why the item is not applicable/relevant for your study

No

## 7a) How sample size was determined

NPT: When applicable, details of whether and how the clustering by care provides or centers was addressed

**7a-i) Describe whether and how expected attrition was taken into account when calculating the sample size**

Describe whether and how expected attrition was taken into account when calculating the sample size.

| | 1 | 2 | 3 | 4 | 5 | |
|---|---|---|---|---|---|---|
| subitem not at all important | ○ | ○ | ○ | ○ | ○ | essential |

**Does your paper address subitem 7a-i?**

Copy and paste relevant sections from manuscript title (include quotes in quotation marks "like this" to indicate direct quotes from your manuscript), or elaborate on this item by providing additional information not in the ms, or briefly explain why the item is not applicable/relevant for your study

# 7b) When applicable, explanation of any interim analyses and stopping guidelines

**Does your paper address CONSORT subitem 7b? ***

Copy and paste relevant sections from the manuscript (include quotes in quotation marks "like this" to indicate direct quotes from your manuscript), or elaborate on this item by providing additional information not in the ms, or briefly explain why the item is not applicable/relevant for your study

Not addressed in manuscript. Level of depressive symptoms (including suicidal thought/level) monitored weekly by self assessment form.

# 8a) Method used to generate the random allocation sequence

NPT: When applicable, how care providers were allocated to each trial group

**Does your paper address CONSORT subitem 8a? ***

Copy and paste relevant sections from the manuscript (include quotes in quotation marks "like this" to indicate direct quotes from your manuscript), or elaborate on this item by providing additional information not in the ms, or briefly explain why the item is not applicable/relevant for your study

"Randomization was performed by a person blinded to screening and baseline data using Stata v.13 proc Ralloc with a block size of two."

# 8b) Type of randomisation; details of any restriction (such as blocking and block size)

**Does your paper address CONSORT subitem 8b? ***

Copy and paste relevant sections from the manuscript (include quotes in quotation marks "like this" to indicate direct quotes from your manuscript), or elaborate on this item by providing additional information not in the ms, or briefly explain why the item is not applicable/relevant for your study

"Randomization was performed by a person blinded to screening and baseline data using Stata v.13 proc Ralloc with a block size of two."

# 9) Mechanism used to implement the random allocation sequence (such as sequentially numbered containers), describing any steps taken to conceal the sequence until interventions were assigned

**Does your paper address CONSORT subitem 9? ***

Copy and paste relevant sections from the manuscript (include quotes in quotation marks "like this" to indicate direct quotes from your manuscript), or elaborate on this item by providing additional information not in the ms, or briefly explain why the item is not applicable/relevant for your study

List with study id-number of included participants (with out any other information) sent to blinded person performing randomization. "Randomization was performed by a person blinded to screening and baseline data using Stata v.13 proc Ralloc with a block size of two."

# 10) Who generated the random allocation sequence, who enrolled participants, and who assigned participants to interventions

**Does your paper address CONSORT subitem 10? ***

Copy and paste relevant sections from the manuscript (include quotes in quotation marks "like this" to indicate direct quotes from your manuscript), or elaborate on this item by providing additional information not in the ms, or briefly explain why the item is not applicable/relevant for your study

Random allocation sequence generated by blinded statistician, enrollment preformed by the first author (JL), assignment performed by last author (PJ) who at that time was blinded to participants identity, study id and, screening and baseline data.

# 11a) If done, who was blinded after assignment to interventions (for example, participants, care providers, those assessing outcomes) and how

NPT: Whether or not administering co-interventions were blinded to group assignment

**11a-i) Specify who was blinded, and who wasn't**

Specify who was blinded, and who wasn't. Usually, in web-based trials it is not possible to blind the participants [1, 3] (this should be clearly acknowledged), but it may be possible to blind outcome assessors, those doing data analysis or those administering co-interventions (if any).

1 2 3 4 5

subitem not at all important ○ ○ ◉ ○ ○ essential

**Does your paper address subitem 11a-i? ***

Copy and paste relevant sections from the manuscript (include quotes in quotation marks "like this" to indicate direct quotes from your manuscript), or elaborate on this item by providing additional information not in the ms, or briefly explain why the item is not applicable/relevant for your study

No blinding after intervention had commenced.

**11a-ii) Discuss e.g., whether participants knew which intervention was the "intervention of interest" and which one was the "comparator"**

Informed consent procedures (4a-ii) can create biases and certain expectations - discuss e.g., whether participants knew which intervention was the "intervention of interest" and which one was the "comparator".

1 2 3 4 5

subitem not at all important ○ ○ ◉ ○ ○ essential

**Does your paper address subitem 11a-ii?**

Copy and paste relevant sections from the manuscript (include quotes in quotation marks "like this" to indicate direct quotes from your manuscript), or elaborate on this item by providing additional information not in the ms, or briefly explain why the item is not applicable/relevant for your study

Not clearly addressed in the manuscript. However the information given (written and verbal) given to participants prior to informed consent was obtained stated that the purpose of the study was to investigate the effect of ICBT in patients with heart failure and depressive symptoms.

# 11b) If relevant, description of the similarity of interventions

(this item is usually not relevant for ehealth trials as it refers to similarity of a placebo or sham intervention to a active medication/intervention)

**Does your paper address CONSORT subitem 11b? ***

Copy and paste relevant sections from the manuscript (include quotes in quotation marks "like this" to indicate direct quotes from your manuscript), or elaborate on this item by providing additional information not in the ms, or briefly explain why the item is not applicable/relevant for your study

Both intervention (ICBT) and control (moderated discussion forum) were performed thru a web-based platform, requiring the same log-in procedure. Assessments (self-reported online questionnaires and telephone interview at inclusion) was identical. Reminders if assessment forms were not completed were identical. Both interventions regarded the same health problems (heart failure and depressive symptoms).

## 12a) Statistical methods used to compare groups for primary and secondary outcomes

NPT: When applicable, details of whether and how the clustering by care providers or centers was addressed

**Does your paper address CONSORT subitem 12a? ***

Copy and paste relevant sections from the manuscript (include quotes in quotation marks "like this" to indicate direct quotes from your manuscript), or elaborate on this item by providing additional information not in the ms, or briefly explain why the item is not applicable/relevant for your study

"Analysis of participants' characteristics was performed with descriptive statistics (mean, standard deviation, percent and frequencies). Differences in continuous variables were analyzed with a t-test. A chi-square test was used for nominal data, and if the expected number of observations was less than five, Fisher´s exact test was used. Data was checked for assumptions of normality and primary outcome measurements were found suitable for parametric analysis. All analysis were [illegible]

### 12a-i) Imputation techniques to deal with attrition / missing values

Imputation techniques to deal with attrition / missing values: Not all participants will use the intervention/comparator as intended and attrition is typically high in ehealth trials. Specify how participants who did not use the application or dropped out from the trial were treated in the statistical analysis (a complete case analysis is strongly discouraged, and simple imputation techniques such as LOCF may also be problematic [4]).

| | 1 | 2 | 3 | 4 | 5 | |
|---|---|---|---|---|---|---|
| subitem not at all important | ○ | ○ | ◉ | ○ | ○ | essential |

**Does your paper address subitem 12a-i? ***

Copy and paste relevant sections from the manuscript (include quotes in quotation marks "like this" to indicate direct quotes from your manuscript), or elaborate on this item by providing additional information not in the ms, or briefly explain why the item is not applicable/relevant for your study

"A total of 18.4% of the participants had missing data at the 9W follow-up measurement. Missing values analysis was performed and data missing completely at random (MCAR) was assumed since there were no significant differences between background variables for participants with complete data versus incomplete data, and Little´s test for MCAR was not significant (  2(111, N = 50) =82.07, P=.98). Missing values were imputed using the expectation-maximization (EM) [illegible]

## 12b) Methods for additional analyses, such as subgroup analyses and adjusted analyses

**Does your paper address CONSORT subitem 12b? ***

Copy and paste relevant sections from the manuscript (include quotes in quotation marks "like this" to indicate direct quotes from your manuscript), or elaborate on this item by providing additional information not in the ms, or briefly explain why the item is not applicable/relevant for your study

"Subgroup analysis was performed on participants with complete data."

## X26) REB/IRB Approval and Ethical Considerations [recommended as subheading under "Methods"] (not a CONSORT item)

**X26-i) Comment on ethics committee approval**

| | 1 | 2 | 3 | 4 | 5 | |
|---|---|---|---|---|---|---|
| subitem not at all important | ○ | ○ | ○ | ◉ | ○ | essential |

**Does your paper address subitem X26-i?**

Copy and paste relevant sections from the manuscript (include quotes in quotation marks "like this" to indicate direct quotes from your manuscript), or elaborate on this item by providing additional information not in the ms, or briefly explain why the item is not applicable/relevant for your study

"The regional ethical review board in Linköping, Sweden approved the study."

**x26-ii) Outline informed consent procedures**

Outline informed consent procedures e.g., if consent was obtained offline or online (how? Checkbox, etc.?), and what information was provided (see 4a-ii). See [6] for some items to be included in informed consent documents.

| | 1 | 2 | 3 | 4 | 5 | |
|---|---|---|---|---|---|---|
| subitem not at all important | ○ | ○ | ◉ | ○ | ○ | essential |

**Does your paper address subitem X26-ii?**

Copy and paste relevant sections from the manuscript (include quotes in quotation marks "like this" to indicate direct quotes from your manuscript), or elaborate on this item by providing additional information not in the ms, or briefly explain why the item is not applicable/relevant for your study

"All participants gave written informed consent."
Informed consent was obtained both online (when participants registered there interest for participation in the study, by checkbox) and offline by mailing a signed consent form after the telephone interview (only participants that was not excluded before this stage in the inclusion process).

**X26-iii) Safety and security procedures**

Safety and security procedures, incl. privacy considerations, and any steps taken to reduce the likelihood or detection of harm (e.g., education and training, availability of a hotline)

| | 1 | 2 | 3 | 4 | 5 | |
|---|---|---|---|---|---|---|
| subitem not at all important | ○ | ○ | ○ | ◉ | ○ | essential |

**Does your paper address subitem X26-iii?**

Copy and paste relevant sections from the manuscript (include quotes in quotation marks "like this" to indicate direct quotes from your manuscript), or elaborate on this item by providing additional information not in the ms, or briefly explain why the item is not applicable/relevant for your study

"Each participant received a password and an unidentifiable username. Login to the web-portal (to access the treatment program, feedback, secure e-mail, and assessment forms) required a two-factor authentication system to protect sensitive information. If technical problems occurred, both the therapist and the participants could get support from a computer technician."

# RESULTS

## 13a) For each group, the numbers of participants who were randomly assigned, received intended treatment, and were analysed for the primary outcome

NPT: The number of care providers or centers performing the intervention in each group and the number of patients treated by each care provider in each center

**Does your paper address CONSORT subitem 13a? ***

Copy and paste relevant sections from the manuscript (include quotes in quotation marks "like this" to indicate direct quotes from your manuscript), or elaborate on this item by providing additional information not in the ms, or briefly explain why the item is not applicable/relevant for your study

Yes see figure 1 (flowchart) and table 2 and: "Mean values for PHQ-9 at baseline and 9W follow-up in the two groups (n=25 ICBT and n=25 DF)" "Change in cardiac anxiety (CAQ) – mean values for total score and subscales in the two groups (n=25 ICBT and n=25 DF)" "Change in Minnesota Living with Heart Failure (MLHF) – total score and factors in the two groups (n=25 ICBT and n=25 DF)"

## 13b) For each group, losses and exclusions after randomisation, together with reasons

**Does your paper address CONSORT subitem 13b? (NOTE: Preferably, this is shown in a CONSORT flow diagram) ***

Copy and paste relevant sections from the manuscript (include quotes in quotation marks "like this" to indicate direct quotes from your manuscript), or elaborate on this item by providing additional information not in the ms, or briefly explain why the item is not applicable/relevant for your study

Yes se figure 1 (flowchart)

### 13b-i) Attrition diagram

Strongly recommended: An attrition diagram (e.g., proportion of participants still logging in or using the intervention/comparator in each group plotted over time, similar to a survival curve) or other figures or tables demonstrating usage/dose/engagement.

| | 1 | 2 | 3 | 4 | 5 | |
|---|---|---|---|---|---|---|
| subitem not at all important | ○ | ○ | ◉ | ○ | ○ | essential |

**Does your paper address subitem 13b-i?**

Copy and paste relevant sections from the manuscript or cite the figure number if applicable (include quotes in quotation marks "like this" to indicate direct quotes from your manuscript), or elaborate on this item by providing additional information not in the ms, or briefly explain why the item is not applicable/relevant for your study

No diagram, information given in text.

## 14a) Dates defining the periods of recruitment and follow-up

**Does your paper address CONSORT subitem 14a? ***

Copy and paste relevant sections from the manuscript (include quotes in quotation marks "like this" to indicate direct quotes from your manuscript), or elaborate on this item by providing additional information not in the ms, or briefly explain why the item is not applicable/relevant for your study

"To recruit participants an information letter was sent to all patients who had an outpatient appointment or who had been admitted to hospital with the main diagnosis of HF during 2013 and 2014"

**14a-i) Indicate if critical "secular events" fell into the study period**

Indicate if critical "secular events" fell into the study period, e.g., significant changes in Internet resources available or "changes in computer hardware or Internet delivery resources"

| | 1 | 2 | 3 | 4 | 5 | |
|---|---|---|---|---|---|---|
| subitem not at all important | ○ | ○ | ◉ | ○ | ○ | essential |

**Does your paper address subitem 14a-i?**

Copy and paste relevant sections from the manuscript (include quotes in quotation marks "like this" to indicate direct quotes from your manuscript), or elaborate on this item by providing additional information not in the ms, or briefly explain why the item is not applicable/relevant for your study

N/A

## 14b) Why the trial ended or was stopped (early)

**Does your paper address CONSORT subitem 14b? ***

Copy and paste relevant sections from the manuscript (include quotes in quotation marks "like this" to indicate direct quotes from your manuscript), or elaborate on this item by providing additional information not in the ms, or briefly explain why the item is not applicable/relevant for your study

N/A

# 15) A table showing baseline demographic and clinical characteristics for each group

NPT: When applicable, a description of care providers (case volume, qualification, expertise, etc.) and centers (volume) in each group

**Does your paper address CONSORT subitem 15? ***

Copy and paste relevant sections from the manuscript (include quotes in quotation marks "like this" to indicate direct quotes from your manuscript), or elaborate on this item by providing additional information not in the ms, or briefly explain why the item is not applicable/relevant for your study

Yes, see table 2.

**15-i) Report demographics associated with digital divide issues**

In ehealth trials it is particularly important to report demographics associated with digital divide issues, such as age, education, gender, social-economic status, computer/Internet/ehealth literacy of the participants, if known.

| | 1 | 2 | 3 | 4 | 5 | |
|---|---|---|---|---|---|---|
| subitem not at all important | ○ | ○ | ○ | ◉ | ○ | essential |

**Does your paper address subitem 15-i? ***

Copy and paste relevant sections from the manuscript (include quotes in quotation marks "like this" to indicate direct quotes from your manuscript), or elaborate on this item by providing additional information not in the ms, or briefly explain why the item is not applicable/relevant for your study

Age and gender reported.

# 16) For each group, number of participants (denominator) included in each analysis and whether the analysis was by original assigned groups

**16-i) Report multiple “denominators” and provide definitions**

Report multiple “denominators” and provide definitions: Report N’s (and effect sizes) “across a range of study participation [and use] thresholds” [1], e.g., N exposed, N consented, N used more than x times, N used more than y weeks, N participants “used” the

intervention/comparator at specific pre-defined time points of interest (in absolute and relative numbers per group). Always clearly define "use" of the intervention.

| | 1 | 2 | 3 | 4 | 5 | |
|---|---|---|---|---|---|---|
| subitem not at all important | ○ | ○ | ○ | ○ | ○ | essential |

**Does your paper address subitem 16-i? ***

Copy and paste relevant sections from the manuscript (include quotes in quotation marks "like this" to indicate direct quotes from your manuscript), or elaborate on this item by providing additional information not in the ms, or briefly explain why the item is not applicable/relevant for your study

"A separate analysis of participants with PHQ-9 ≥10 at screening (ICBT n=18, DF n=15)" "ICBT group and women (n=8 (complete cases))" "compared to men (n=13 (complete cases)) "

**16-ii) Primary analysis should be intent-to-treat**

Primary analysis should be intent-to-treat, secondary analyses could include comparing only "users", with the appropriate caveats that this is no longer a randomized sample (see 18-i).

| | 1 | 2 | 3 | 4 | 5 | |
|---|---|---|---|---|---|---|
| subitem not at all important | ○ | ○ | ○ | ◉ | ○ | essential |

**Does your paper address subitem 16-ii?**

Copy and paste relevant sections from the manuscript (include quotes in quotation marks "like this" to indicate direct quotes from your manuscript), or elaborate on this item by providing additional information not in the ms, or briefly explain why the item is not applicable/relevant for your study

"All analysis were performed according to the intention to treat principle regardless of actual completion of the ICBT program or DF."

# 17a) For each primary and secondary outcome, results for each group, and the estimated effect size and its precision (such as 95% confidence interval)

**Does your paper address CONSORT subitem 17a? ***

Copy and paste relevant sections from the manuscript (include quotes in quotation marks "like this" to indicate direct quotes from your manuscript), or elaborate on this item by providing

additional information not in the ms, or briefly explain why the item is not applicable/relevant for your study

"Depressive symptoms in patients in the ICBT group decreased significantly from baseline to the 9W follow-up (Figure 3) (M = 10.8 SD±5.7 vs. M = 8.6 SD±4.6, t(24)=2.6, P=.02. Cohen´s d=0.43)."

### 17a-i) Presentation of process outcomes such as metrics of use and intensity of use

In addition to primary/secondary (clinical) outcomes, the presentation of process outcomes such as metrics of use and intensity of use (dose, exposure) and their operational definitions is critical. This does not only refer to metrics of attrition (13-b) (often a binary variable), but also to more continuous exposure metrics such as "average session length". These must be accompanied by a technical description how a metric like a "session" is defined (e.g., timeout after idle time) [1] (report under item 6a).

| | 1 | 2 | 3 | 4 | 5 | |
|---|---|---|---|---|---|---|
| subitem not at all important | ○ | ○ | ○ | ○ | ○ | essential |

**Does your paper address subitem 17a-i?**

Copy and paste relevant sections from the manuscript (include quotes in quotation marks "like this" to indicate direct quotes from your manuscript), or elaborate on this item by providing additional information not in the ms, or briefly explain why the item is not applicable/relevant for your study

"The median number of modules performed in the ICBT group was four. Six of the participants in the ICBT group had worked with all seven modules and 15 (60%) had worked with at least four modules." In the ICBT group, the number of logins to the web-portal was" "Activity in the program was calculated by the number of modules that the participants worked with (ICBT group only) as well as the number of logins to the web-portal during the nine-week period (both groups)."

## 17b) For binary outcomes, presentation of both absolute and relative effect sizes is recommended

**Does your paper address CONSORT subitem 17b? ***

Copy and paste relevant sections from the manuscript (include quotes in quotation marks "like this" to indicate direct quotes from your manuscript), or elaborate on this item by providing additional information not in the ms, or briefly explain why the item is not applicable/relevant for your study

N/A no binary outcomes.

## 18) Results of any other analyses performed, including subgroup analyses and adjusted analyses, distinguishing pre-specified from exploratory

**Does your paper address CONSORT subitem 18? ***

Copy and paste relevant sections from the manuscript (include quotes in quotation marks "like this" to indicate direct quotes from your manuscript), or elaborate on this item by providing additional information not in the ms, or briefly explain why the item is not applicable/relevant for your study

"Subgroup analysis was performed on participants with complete data."

**18-i) Subgroup analysis of comparing only users**

A subgroup analysis of comparing only users is not uncommon in ehealth trials, but if done, it must be stressed that this is a self-selected sample and no longer an unbiased sample from a randomized trial (see 16-iii).

| | 1 | 2 | 3 | 4 | 5 | |
|---|---|---|---|---|---|---|
| subitem not at all important | ○ | ○ | ○ | ◉ | ○ | essential |

**Does your paper address subitem 18-i?**

Copy and paste relevant sections from the manuscript (include quotes in quotation marks "like this" to indicate direct quotes from your manuscript), or elaborate on this item by providing additional information not in the ms, or briefly explain why the item is not applicable/relevant for your study

"Subgroup analysis was performed on participants with complete data."

## 19) All important harms or unintended effects in each group

(for specific guidance see CONSORT for harms)

**Does your paper address CONSORT subitem 19? ***

Copy and paste relevant sections from the manuscript (include quotes in quotation marks "like this" to indicate direct quotes from your manuscript), or elaborate on this item by providing additional information not in the ms, or briefly explain why the item is not applicable/relevant for your study

Yes, no harms or unintended effects reported to or know by the research team.

**19-i) Include privacy breaches, technical problems**

Include privacy breaches, technical problems. This does not only include physical "harm" to participants, but also incidents such as perceived or real privacy breaches [1], technical problems, and other unexpected/unintended incidents. "Unintended effects" also includes unintended positive effects [2].

| | 1 | 2 | 3 | 4 | 5 | |
|---|---|---|---|---|---|---|
| subitem not at all important | ○ | ○ | ○ | ◉ | ○ | essential |

**Does your paper address subitem 19-i?**

Copy and paste relevant sections from the manuscript (include quotes in quotation marks "like this" to indicate direct quotes from your manuscript), or elaborate on this item by providing additional information not in the ms, or briefly explain why the item is not applicable/relevant for your study

No privacy breaches reported or known to the research team. No major technical problems reported (individual participants had minor technical problems that were solved either by information from the therapist or by support from the computer technician with in a few hours to one or two days).

**19-ii) Include qualitative feedback from participants or observations from staff/researchers**

Include qualitative feedback from participants or observations from staff/researchers, if available, on strengths and shortcomings of the application, especially if they point to unintended/unexpected effects or uses. This includes (if available) reasons for why people did or did not use the application as intended by the developers.

| | 1 | 2 | 3 | 4 | 5 | |
|---|---|---|---|---|---|---|
| subitem not at all important | ○ | ○ | ○ | ◉ | ○ | essential |

**Does your paper address subitem 19-ii?**

Copy and paste relevant sections from the manuscript (include quotes in quotation marks "like this" to indicate direct quotes from your manuscript), or elaborate on this item by providing additional information not in the ms, or briefly explain why the item is not applicable/relevant for your study

An important part of the evaluation of a intervention. In our manuscript this is not covered because we intend to present a separate manuscript on this with an in-depth qualitative analysis of the participants experiences (data collected and analysis in progress).

# DISCUSSION

## 22) Interpretation consistent with results, balancing benefits and harms, and considering other relevant evidence

NPT: In addition, take into account the choice of the comparator, lack of or partial blinding, and unequal expertise of care providers or centers in each group

### 22-i) Restate study questions and summarize the answers suggested by the data, starting with primary outcomes and process outcomes (use)

Restate study questions and summarize the answers suggested by the data, starting with primary outcomes and process outcomes (use).

| | 1 | 2 | 3 | 4 | 5 | |
|---|---|---|---|---|---|---|
| subitem not at all important | ○ | ○ | ◉ | ○ | ○ | essential |

**Does your paper address subitem 22-i? ***

Copy and paste relevant sections from the manuscript (include quotes in quotation marks "like this" to indicate direct quotes from your manuscript), or elaborate on this item by providing additional information not in the ms, or briefly explain why the item is not applicable/relevant for your study

"We found that after using a guide self-help ICBT program patients depressive symptoms and fear was reduced. The results are encouraging in the sense that the depressive symptoms were significantly lower at the 9W follow-up compared to baseline. However, the between group analysis only showed non-significant tendencies in favor of the ICBT group. The lack of significant difference might partly be explained by the slight reduction of depressive symptoms in [illegible]

### 22-ii) Highlight unanswered new questions, suggest future research

Highlight unanswered new questions, suggest future research.

| | 1 | 2 | 3 | 4 | 5 | |
|---|---|---|---|---|---|---|
| subitem not at all important | ○ | ○ | ◉ | ○ | ○ | essential |

**Does your paper address subitem 22-ii?**

Copy and paste relevant sections from the manuscript (include quotes in quotation marks "like this" to indicate direct quotes from your manuscript), or elaborate on this item by providing

additional information not in the ms, or briefly explain why the item is not applicable/relevant for your study

"The effect of our ICBT program, focusing mainly on depressive symptoms, may therefore be improved by also adding CBT components that focus on worries and anxiety." "Our ICBT program relied to a large extent on behavioral activation and to a lesser extent on problem-solving therapy [17]. The majority of patients with HF are older and vulnerable, which raises an important question of whether problem-solving therapy should be used to a greater extent in [illegible]

# 20) Trial limitations, addressing sources of potential bias, imprecision, and, if relevant, multiplicity of analyses

### 20-i) Typical limitations in ehealth trials

Typical limitations in ehealth trials: Participants in ehealth trials are rarely blinded. Ehealth trials often look at a multiplicity of outcomes, increasing risk for a Type I error. Discuss biases due to non-use of the intervention/usability issues, biases through informed consent procedures, unexpected events.

| | 1 | 2 | 3 | 4 | 5 | |
|---|---|---|---|---|---|---|
| subitem not at all important | ○ | ○ | ◉ | ○ | ○ | essential |

### Does your paper address subitem 20-i? *

Copy and paste relevant sections from the manuscript (include quotes in quotation marks "like this" to indicate direct quotes from your manuscript), or elaborate on this item by providing additional information not in the ms, or briefly explain why the item is not applicable/relevant for your study

"A major limitation of our study is that it is underpowered. Recruitment of participants was more difficult than expected" "With our study's limitation of sample size and its recruitment difficulties, the results may best serve as a foundation for further research rather than as clinical recommendations."

# 21) Generalisability (external validity, applicability) of the trial findings

NPT: External validity of the trial findings according to the intervention, comparators, patients, and care providers or centers involved in the trial

### 21-i) Generalizability to other populations

Generalizability to other populations: In particular, discuss generalizability to a general Internet population, outside of a RCT setting, and general patient population, including applicability of the study results for other organizations

| | 1 | 2 | 3 | 4 | 5 | |
|---|---|---|---|---|---|---|
| subitem not at all important | ○ | ○ | ◉ | ○ | ○ | essential |

**Does your paper address subitem 21-i?**

Copy and paste relevant sections from the manuscript (include quotes in quotation marks "like this" to indicate direct quotes from your manuscript), or elaborate on this item by providing additional information not in the ms, or briefly explain why the item is not applicable/relevant for your study

"With our study's limitation of sample size and its recruitment difficulties, the results may best serve as a foundation for further research rather than as clinical recommendations."

**21-ii) Discuss if there were elements in the RCT that would be different in a routine application setting**

Discuss if there were elements in the RCT that would be different in a routine application setting (e.g., prompts/reminders, more human involvement, training sessions or other co-interventions) and what impact the omission of these elements could have on use, adoption, or outcomes if the intervention is applied outside of a RCT setting.

| | 1 | 2 | 3 | 4 | 5 | |
|---|---|---|---|---|---|---|
| subitem not at all important | ○ | ○ | ○ | ○ | ○ | essential |

**Does your paper address subitem 21-ii?**

Copy and paste relevant sections from the manuscript (include quotes in quotation marks "like this" to indicate direct quotes from your manuscript), or elaborate on this item by providing additional information not in the ms, or briefly explain why the item is not applicable/relevant for your study

N/A more research on the study area is needed before elaborating on this.

# OTHER INFORMATION

# 23) Registration number and name of trial registry

**Does your paper address CONSORT subitem 23?** *

Copy and paste relevant sections from the manuscript (include quotes in quotation marks "like this" to indicate direct quotes from your manuscript), or elaborate on this item by providing additional information not in the ms, or briefly explain why the item is not applicable/relevant for your study

"The study is registered at clinicaltrails.gov (NCT01681771)."

## 24) Where the full trial protocol can be accessed, if available

**Does your paper address CONSORT subitem 24? ***

Cite a Multimedia Appendix, other reference, or copy and paste relevant sections from the manuscript (include quotes in quotation marks "like this" to indicate direct quotes from your manuscript), or elaborate on this item by providing additional information not in the ms, or briefly explain why the item is not applicable/relevant for your study

Yes by contact with the research team

## 25) Sources of funding and other support (such as supply of drugs), role of funders

**Does your paper address CONSORT subitem 25? ***

Copy and paste relevant sections from the manuscript (include quotes in quotation marks "like this" to indicate direct quotes from your manuscript), or elaborate on this item by providing additional information not in the ms, or briefly explain why the item is not applicable/relevant for your study

## X27) Conflicts of Interest (not a CONSORT item)

**X27-i) State the relation of the study team towards the system being evaluated**

In addition to the usual declaration of interests (financial or otherwise), also state the relation of the study team towards the system being evaluated, i.e., state if the authors/evaluators are distinct from or identical with the developers/sponsors of the intervention.

1 2 3 4 5

subitem not at all important ○ ○ ○ ○ ○ essential

**Does your paper address subitem X27-i?**

Copy and paste relevant sections from the manuscript (include quotes in quotation marks "like this" to indicate direct quotes from your manuscript), or elaborate on this item by providing additional information not in the ms, or briefly explain why the item is not applicable/relevant for your study

"Funding sources
This study was funded by grants from the Swedish Heart and Lung Association (grant number E087/13 and E08/14), the Medical Research Council of Southeast Sweden (grant numbers FORSS-374721 and FORSS-470121) and the Region Östergötland (grant numbers LIO-355611, LIO-374831, LIO-443711 and LIO-470271). The funding sources had no influence on the design, procedure, analysis or interpretation of [illegible]

# About the CONSORT EHEALTH checklist

**As a result of using this checklist, did you make changes in your manuscript?** *

○ yes, major changes

◉ yes, minor changes

○ no

**What were the most important changes you made as a result of using this checklist?**

**How much time did you spend on going through the checklist INCLUDING making changes in your manuscript** *

3 hours

**As a result of using this checklist, do you think your manuscript has improved?** *

◉ yes

○ no

○ Övrigt:

**Would you like to become involved in the CONSORT EHEALTH group?**

This would involve for example becoming involved in participating in a workshop and writing an "Explanation and Elaboration" document

○ yes

◉ no

○ Övrigt: [ ]

**Any other comments or questions on CONSORT EHEALTH**

## STOP - Save this form as PDF before you click submit

To generate a record that you filled in this form, we recommend to generate a PDF of this page (on a Mac, simply select "print" and then select "print as PDF") before you submit it.

When you submit your (revised) paper to JMIR, please upload the PDF as supplementary file.

Don't worry if some text in the textboxes is cut off, as we still have the complete information in our database. Thank you!

## Final step: Click submit !

Click submit so we have your answers in our database!

Skicka

*Skicka aldrig lösenord med Google Formulär*

Tillhandahålls av

Det här innehållet har varken skapats eller godkänts av Google.
Anmäl otillåten användning - Användarvillkor - Ytterligare villkor
